# Fitness Landscape-Guided Engineering of Locally Supercharged Virus-like Particles with Enhanced Cell Uptake Properties

**DOI:** 10.1021/acschembio.2c00318

**Published:** 2022-11-15

**Authors:** Paige E. Pistono, Paul Huang, Daniel D. Brauer, Matthew B. Francis

**Affiliations:** † Department of Chemistry, University of California, Berkeley, California94720, United States; ‡ Materials Sciences Division, Lawrence Berkeley National Laboratory, Berkeley, California94720, United States

## Abstract

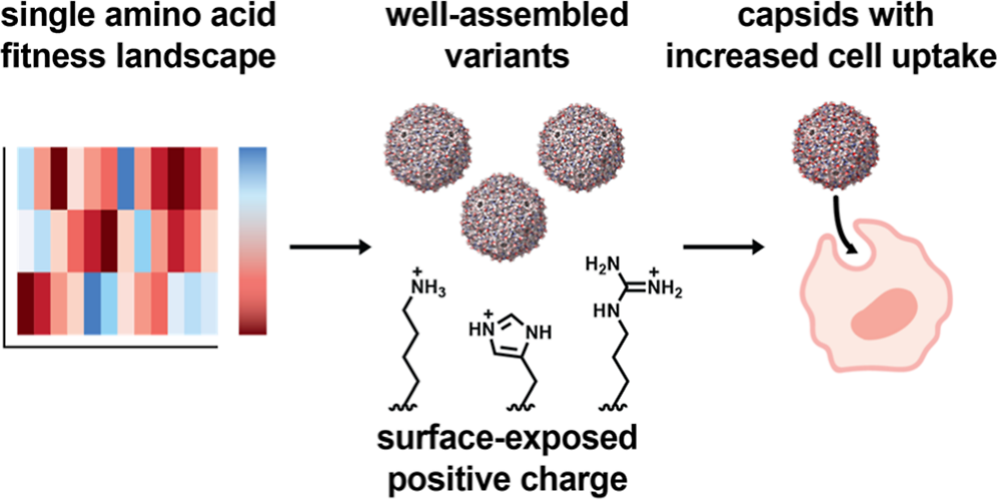

Protein-based nanoparticles are useful models for the study of self-assembly and attractive candidates for drug delivery. Virus-like particles (VLPs) are especially promising platforms for expanding the repertoire of therapeutics that can be delivered effectively as they can deliver many copies of a molecule per particle for each delivery event. However, their use is often limited due to poor uptake of VLPs into mammalian cells. In this study, we use the fitness landscape of the bacteriophage MS2 VLP as a guide to engineer capsid variants with positively charged surface residues to enhance their uptake into mammalian cells. By combining mutations with positive fitness scores that were likely to produce assembled capsids, we identified two key double mutants with internalization efficiencies as much as 67-fold higher than that of wtMS2. Internalization of these variants with positively charged surface residues depends on interactions with cell surface sulfated proteoglycans, and yet, they are biophysically similar to wtMS2 with low cytotoxicity and an overall negative charge. Additionally, the best-performing engineered MS2 capsids can deliver a potent anticancer small-molecule therapeutic with efficacy levels similar to antibody-drug conjugates. Through this work, we were able to establish fitness landscape-based engineering as a successful method for designing VLPs with improved cell penetration. These findings suggest that VLPs with positive surface charge could be useful in improving the delivery of small-molecule- and nucleic acid-based therapeutics.

Engineered nanoparticles (NPs) are promising materials for use in drug delivery, with the potential to enhance cellular uptake, improve cargo stability and solubility, and allow cell-specific targeting.^1^ The most well-characterized nanomaterials for drug delivery include lipid-based and polymeric NPs, with lipid-based NPs being the most common class of FDA-approved nanomedicines.^2^ Here, we discuss the use of a virus-like particle (VLP) as a potential drug delivery vehicle. Unlike polymer- and lipid-based NPs, VLPs can be inexpensively produced by recombinant expression, are homogenous in particle size, and are easily degradable in the body.^3,4^ Protein-based materials can be genetically engineered with new functionalities, and site-specific modification can be achieved through insertion of specific natural or noncanonical amino acids into the protein sequence.^5−7^ Although protein-based NPs possess many attractive features, their exploration has thus far been limited due to poor cellular uptake, with the exception of the use of viral vectors for gene therapy and vaccines.^8^

The utility of engineered, sequence-defined protein NPs with desirable properties would benefit from strategies to fine-tune their surface charge, a key property that dictates their stability and interactions with biological components. In monomeric form, it is well established that cationic peptides and proteins can enter and deliver cargo to mammalian cells, ranging from cell-penetrating peptides such as HIV-Tat to mini cationic proteins and full-sized supercharged proteins.^9−12^ Unlike the prior examples, the mutational effects of installing cationic residues on VLPs would be amplified by the regular periodicity of their nanostructure. We hypothesized that local supercharging on nanoscale particles could result in cellular uptake enhancement with decreased cytotoxicity when compared with commercially available cationic liposomes, thus avoiding a major bottleneck in the application of these NPs for drug and biotherapeutic delivery.^13^ Additionally, expanding the understanding of VLP design rules by altering particle charge could bestow the ability to tune other properties, such as thermostability and pH sensitivity.

Bacteriophage MS2 is a well-studied VLP that forms 27 nm capsids with 2 nm pores and a hollow interior cavity that can be used for cargo loading.^4^ MS2 can be recombinantly expressed as a noninfectious variant at high yields. Importantly, the capsids can encapsulate segments of mRNA during expression and self-assembly, providing a direct genotype-to-phenotype link that can be used for fitness landscaping.^14^ Recently, our lab developed a method known as Systematic Mutation and Assembled Particle Selection (SyMAPS) that was used to characterize the assembly properties of all single amino acid variants of MS2-based VLPs.^15^ Using this method, subsequent fitness landscaping studies of MS2 have identified assembly competent, thermostable, acid-sensitive, and chemically modifiable variants.^15−18^

One property that these fitness landscaping experiments have not selected for is mammalian cell internalization. Previous studies reporting MS2 capsids as delivery vehicles have shown the benefit of targeting groups, such as transferrin, peptides, aptamers, and antibodies that are conjugated to the exterior surfaces of the capsids. Although these strategies are important for enhancing cell binding, some of these studies also noted that MS2 internalization levels were lower than that of other delivery vehicles such as lipid-based NPs.^19−22^ The usefulness of MS2 as a drug delivery vehicle would be expanded immensely by improving its inherent cellular uptake efficiency. With this in mind, we sought to use the single amino acid fitness landscape of MS2 as a guide to design variants that are capable of carrying internal cargo into mammalian cells. We envisioned that we could capitalize on the dense, symmetrical quaternary assembly of MS2 to generate supercharged patches along the capsid surface without significantly shifting the global charge of the particle. This engineering approach would not be possible with other NP systems whose structures are not sequence-defined.

Here, we use fitness landscape-guided engineering to identify well-assembled MS2 variants with positively charged amino acids in specific positions to increase mammalian cell uptake. We identified two key double mutants that produce capsids with localized pockets of positive charge and internalization efficiencies up to 67-fold higher than that of wtMS2. Current evidence suggests that these MS2 variants with positively charged surface residues enter cells through an energy-dependent endocytic pathway that is mediated by binding to sulfated proteoglycans on the cell surface. These engineered variants are biophysically similar to wtMS2, including its negative overall charge, and are nontoxic to cells. We also found that these variants can deliver monomethyl auristatin E (MMAE), a potent anticancer therapeutic, to glioblastoma cells with efficacy comparable to antibody-drug conjugates. These findings indicate that VLPs with strategically placed positive surface charges could overcome key limitations in the delivery of a wide range of small-molecule- and nucleic acid-based therapeutics.

## Results and Discussion

### Design and Characterization of MS2 Variants with Positively Charged Surface Residues

MS2-based VLPs consist of 180 identical copies of the coat protein (CP) in the form of 90 dimers, forming a *T* = 3 icosahedron with several unique interfaces.^14^ Assembly of MS2 VLPs is triggered by a conformational switch from the symmetric C/C dimer to the asymmetric A/B dimer upon binding of a genetic material such as RNA.^23,24^ Binding of a genetic material also provides a direct genotype-to-phenotype link for fitness landscaping, for which our laboratories have developed a technique known as SyMAPS.^15^ SyMAPS was used to characterize the assembly competency of all single amino acid variants of MS2, generating an apparent fitness landscape for the capsid that assigns a quantitative apparent fitness score (AFS) for every variant at every position along the backbone of the MS2 CP. These data thus indicate all of the positions in which new lysine, arginine, and histidine residues can be introduced while still allowing capsid assembly, providing a useful guide for engineering variants with increased cell internalization efficiency.

**Figure 1:**
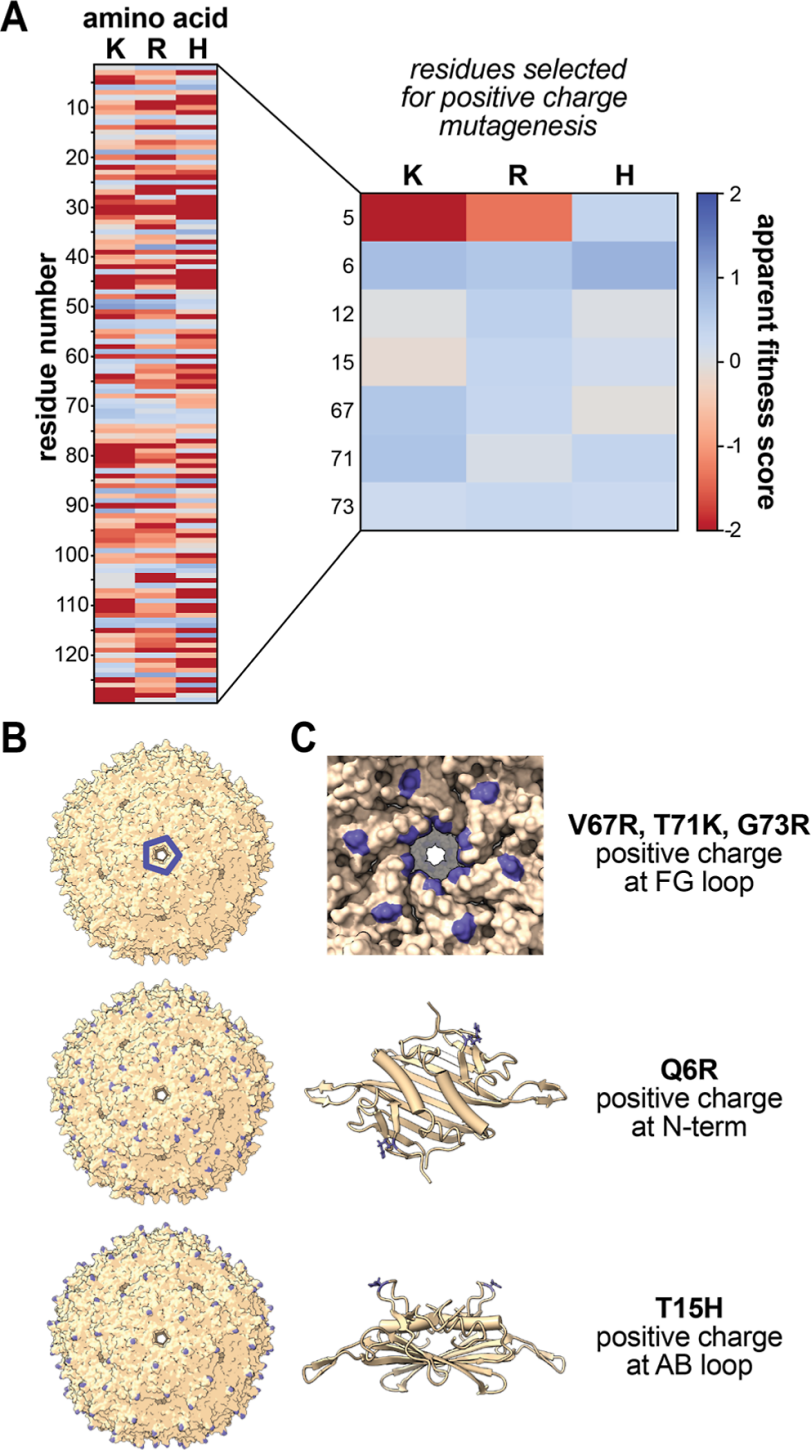
(A) AFSs for all Lys (K), Arg (R), and His (H) variants of the MS2 CP. Positive (blue) AFSs represent increased variant abundance, and negative (red) AFSs correspond to decreased variant abundance. The scale is logarithmic, encompassing a 10,000-fold range of assembly propensity. (B) Locations of positive charge mutations (blue) mapped onto assembled MS2 CP (PDB ID = 2MS2). (C) The locations of positive charge mutations (blue) mapped onto the MS2 CP dimer structure (PDB ID = 2MS2).

Figure 1A shows the AFSs for lysine, arginine, and histidine at every position in the MS2 CP, which were used to select mutations with positive fitness scores that were likely to produce assembled capsids. Histidine mutations were included to probe the possibility of making capsids that would become positively charged in acidic conditions such as the cancer extracellular microenvironment.^25^ Mutations were selected in three different surface-exposed regions of the capsid: the N-terminus (residues 1–6), the AB loop (residues 11–17), and the FG loop (residues 71–76). Mutations near the N-terminus and AB loop would result in an even distribution of positive charges over the capsid surface, while mutations in the FG loop would create localized pockets of positive charge at the capsid pores (Figures 1B,C, S13, and S14). Double and triple mutants were created at the FG loop to probe the effects of concentrated positive charge further. An initial panel of 12 MS2 CP variants containing an interior cysteine (N87C) for bioconjugation was designed and expressed. The assembly state of each variant was confirmed via size exclusion chromatography. Ten of the 12 designed variants were able to produce assembled capsids (Figures S1 and S2). The high success rate of capsid assembly among the 12 MS2 CP variants would not have been possible without fitness landscape-guided design. Out of the 45 surface-exposed residues on the capsid exterior, the assembly success rates for all lysine, arginine, and histidine mutants are 40, 42, and 51%, respectively, compared with an 83% assembly rate for capsid mutations chosen from the MS2 single amino acid fitness landscape.^15^ The successfully assembled MS2 CP variant sequences were also compared to the residues selected for positive charge mutagenesis by Rosetta supercharge, a popular computational method for protein supercharging.^12,26−28^ Among the 45 surface-exposed residues on the capsid exterior, Rosetta supercharge correctly selected an R or K mutation that would result in assembled MS2 capsids with a 70–80% success rate depending on the input structure. Despite this high success rate, Rosetta did not predict two mutations that were selected in this work, N12K and G73R. The MS2 CP fitness landscape contains a wealth of information that cannot always be predicted computationally, such as how mutations will affect quaternary structure properties in the case of capsid assembly. Fitness landscaping can complement these powerful computational tools for protein engineering, serving to further probe the sequence–structure–function relationships underlying closely related proteins.^29−31^

### Identification of MS2 Variants with Enhanced Cellular Uptake

**Figure 2:**
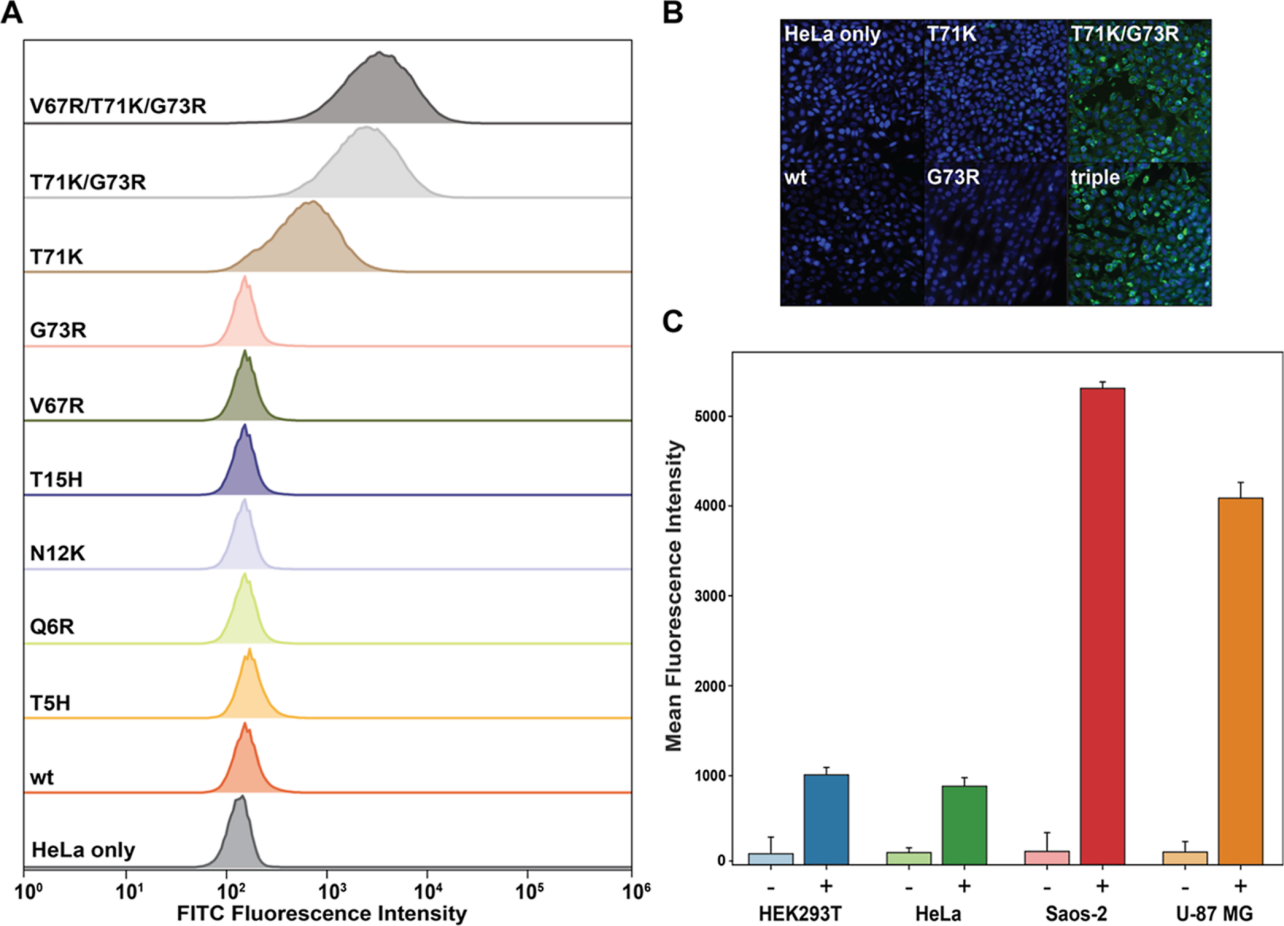
(A) Histograms showing uptake of MS2-fluorescein variants into HeLa cells. Data shown are a concatenation of three biological replicates. (B) Cellular uptake of select MS2-fluorescein variants assessed using fluorescence microscopy. Fixed cell images of HeLa cells treated with 5 μM indicated MS2 CP variants. Triple corresponds to MS2 CP variant V67R/T71K/G73R. (C) Cellular uptake of 5 μM MS2 T71K/G73R-fluorescein into four different cell types [HEK293T (blue), HeLa (green), Saos-2 (red), and U-87 MG (orange)]. Data shown are a concatenation of three biological replicates as measured by flow cytometry. Error bars show the coefficient of variation (CV).

To track their performance in cellular internalization studies, all assembled MS2 capsid variants were labeled with fluorescein-maleimide at the interior cysteine (N87C), and uptake into HeLa cells was screened via fluorescence microscopy and flow cytometry (Figures S3 and S4 and Table S1). Cells were washed with heparin which has been shown to remove positively charged surface-bound species, which we assumed could be useful for removing residual MS2 particles before imaging.^11^ Of the 10 variants analyzed, three variants (MS2 T71K, MS2 T71K/G73R, and MS2 V67R/T71K/G73R) exhibited significantly higher uptake compared to N87C (hereinafter referred to as wtMS2) capsids when visualized with fluorescence microscopy (Figure 2B). The differences in overall uptake were quantified using flow cytometry, with the mean fluorescence intensity (MFI) of HeLa cells treated with MS2 T71K being 4.5-fold higher than that observed for cells treated with wtMS2. Interestingly, combining two single mutations that resulted in low or no internalization into a double mutant, MS2 T71K/G73R, produced an MFI 4-fold higher than cells treated with MS2 T71K and 18-fold higher than cells treated with wtMS2 (Figure 2A and Table S2). Although we were expecting an additive effect with each additional positive charge, the MFI of cells treated with the triple mutant (V67R/T71K/G73R) was only 1.4-fold higher than that of the T71K/G73R double mutant. Thus, the effects of additional positive surface charge on the internalization of MS2 capsids into mammalian cells were both position- and residue-dependent. Previous work on the effects of single mutations on VLP properties found that specific lysine-to-glutamine (K > Q) and lysine-to-glutamate (K > E) point mutants drastically altered Qβ binding to mammalian cells.^32^

**Figure 3:**
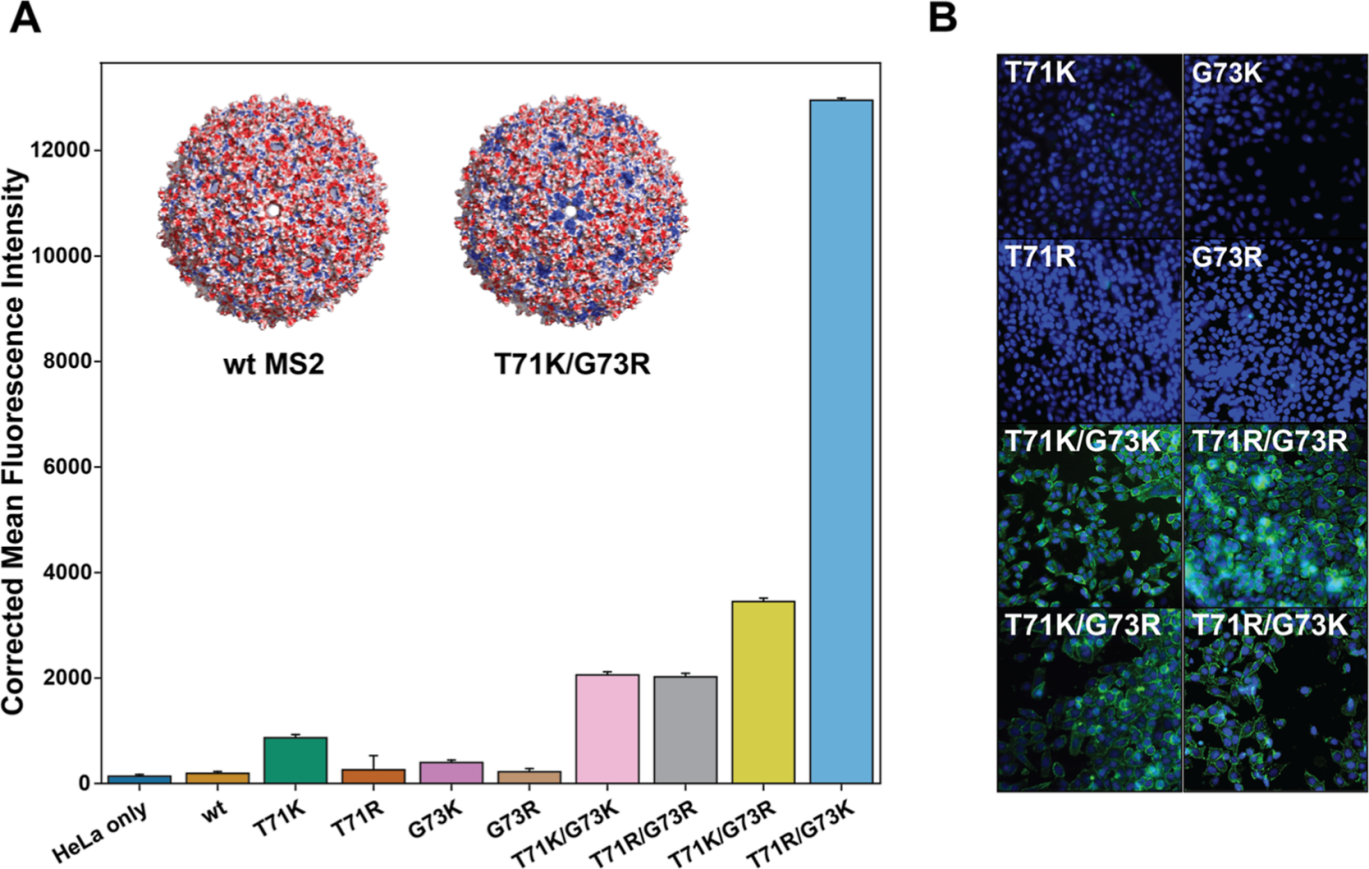
(A) Bar graphs showing corrected MFI of MS2-fluorescein internalization into HeLa cells. Error bars show the CV. Data shown are a concatenation of three biological replicates as measured by flow cytometry. Electrostatic surface potential maps of the wtMS2 and MS2 T71K/G73R capsid are inlaid in the upper portion of the bar graph. Red areas on the structure represent negative charge, while blue areas represent positive surface charge. (B) Cellular uptake of MS2-fluorescein variants with all combinations of positive charge at residues 71 and 73 assessed using fluorescence microscopy. Fixed cell images of HeLa cells treated with 5 μM of the indicated MS2 CP variants.

To explore the position and residue effects on uptake further, we created MS2 capsid variants representing every single and double mutant combination of arginine and lysine at backbone positions 71 and 73, located at the FG loop. This panel of eight MS2 CP variants was expressed, confirmed to assemble, and labeled with fluorescein for internalization experiments (Figures S3 and S4 and Table S1). Imaging showed a visible divide in fluorescence between the single and double mutants, with all four single mutants showing low or no internalization (Figure 3B). Interestingly, variants bearing a combination of arginine and lysine (MS2 T71K/G73R and MS2 T71R/G73K) exhibited significantly higher uptake than double mutants with the same charged residue (KK or RR). We further found that exchanging the position of arginine and lysine (T71K/G73R vs T71R/G73K) resulted in a 3.8-fold enhancement in uptake, yielding a new best-performing variant with a 67-fold enhancement in uptake relative to that of wtMS2 (Figure 3A). Although a previous fitness landscape of double mutants in the FG loop (residues 71–76) region of MS2 provides information on the assembly and thermostability properties of these double mutants, their differences in cellular uptake efficiency could not have been predicted (Table S8).^18^

We compared the internalization of MS2 T71K/G73R into HeLa cells at a range of concentrations and found that punctate fluorescence in the cell interior could still be detected when treating with as low as 2.8 nM assembled capsid particles (0.5 μM MS2 CP monomer concentration) (Figure S9). To test uptake into other cell types of interest, U-87 MG glioblastoma cells, HEK293 embryonic kidney cells, and Saos-2 osteosarcoma cells were also treated with MS2, and internalization was evaluated via confocal microscopy and flow cytometry. Cellular uptake was observed in all four cell types, with 7.6-, 6.5-, 36-, and 29-fold increases in fluorescence intensities in MS2-treated HEK293T, HeLa, Saos-2, and U-87 MG cells, respectively (Figure 2C and Table S3).

### Physical and Biological Characterization of MS2 Variants with Increased Cellular Uptake

**Figure 4:**
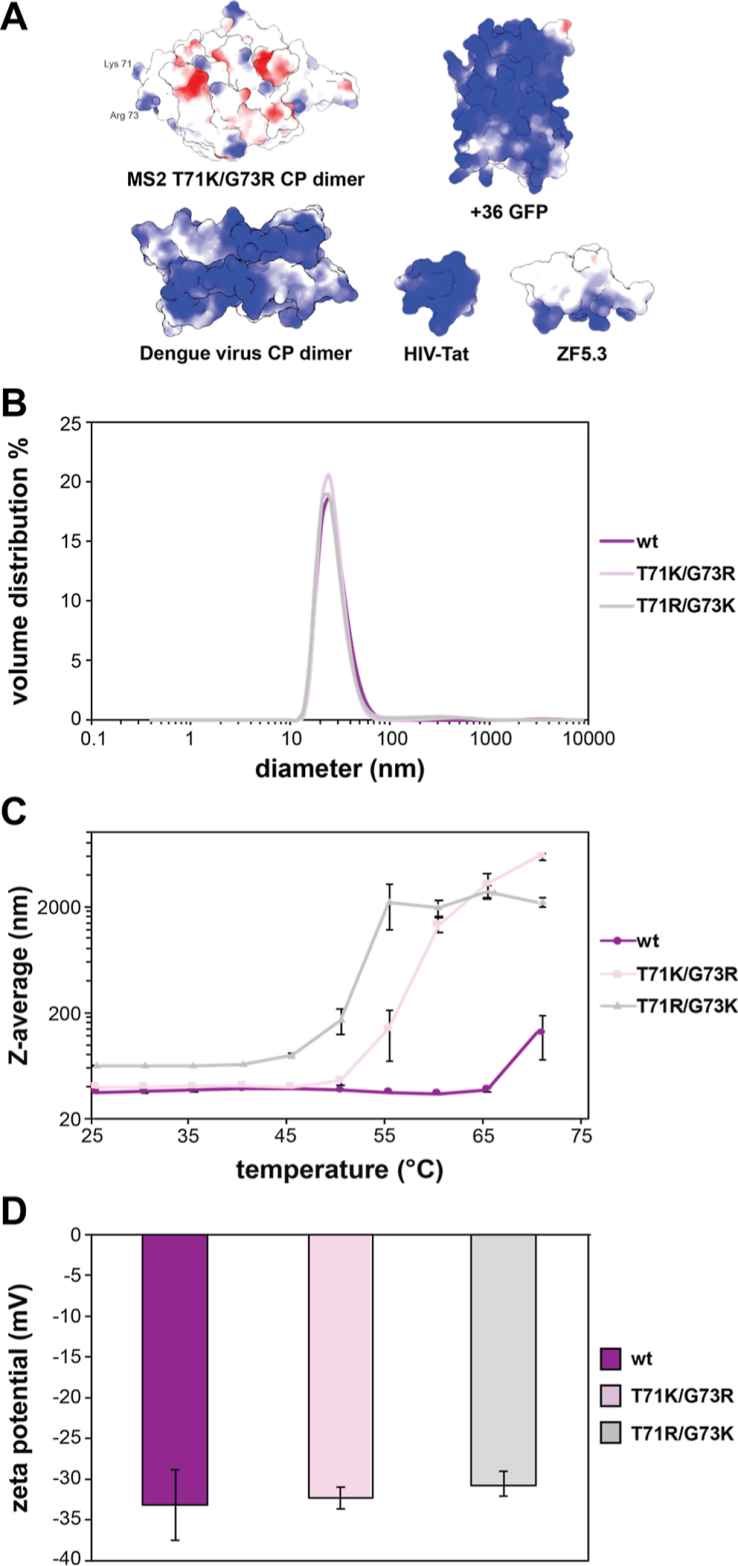
(A) Theoretical electrostatic potential maps of the MS2 T71K/G73R CP dimer exterior face, dengue virus CP dimer exterior face (PDB: 1R6R), +36 GFP (PDB: 2B3P), HIV-Tat (PDB: 1TBC), and ZF5.3 (PDB: 2EOZ). Engineered structures were created using the PyMOL mutagenesis tool. (B) DLS spectra of wtMS2 (purple), MS2 T71K/G73R (pink), and MS2 T71R/G73K (gray) in 10 mM sodium phosphate, pH 7.2. (C) *Z*-average (nm) vs temperature (°C) for wtMS2 (purple circles), MS2 T71K/G73R (pink squares), and MS2 T71R/G73K (gray triangles) in 10 mM sodium phosphate, pH 7.2. Error bars show one standard deviation. (D) Zeta potentials (mV) of wtMS2 (purple), MS2 T71K/G73R (pink), and MS2 T71R/G73K (gray) in 10 mM sodium phosphate, pH 7.2. Error bars show one standard deviation.

The biophysical properties of the top-performing capsids with positively charged surface residues, MS2 T71K/G73R and MS2 T71R/G73K, were evaluated with dynamic light scattering (DLS) and compared with wtMS2. The diameters of all three capsids measured were found to be very similar at 28.2 ± 0.5, 27.0 ± 0.6, and 27.2 ± 0.5 nm for wtMS2, MS2 T71K/G73R, and MS2 T71R/G73K, respectively (Figure 4B). These values are in accordance with literature reports that measured the diameter of wild-type MS2 to be ∼27 nm.^33^ The aggregation point of each variant was measured via a temperature-dependent DLS size screen from 25 to 70 °C. The diameter of wtMS2 was stable until 70 °C, consistent with the previously reported melting temperature of 66 °C (Figure 4C).^34^ The MS2 variants with positively charged surface residues exhibited somewhat lower aggregation points, with MS2 T71K/G73R aggregating at 55 °C and MS2 T71R/G73K aggregating at 50 °C (Figure 4C). We then compared the experimental stability of the MS2 variants to the fitness scores calculated in a previous study in our lab that explored the fitness landscape of all MS2 CP double mutants in the FG loop (residues 71–76).^18^ The trend of calculated thermostability scores for wtMS2, MS2 T71K/G73R, and MS2 T71R/G73K mirrored that of the experimental aggregation points, with a positive thermostability score for wtMS2 (0.42) and negative thermostability scores for MS2 T71K/G73R (−0.29) and MS2 T71R/G73K (−0.54) (Table S8).

Zeta potential measurements were taken for each MS2 variant. These measurements describe the electrostatic potential at the layer of ions bound to the surface of a particle in solution.^35^ The zeta potentials were measured to be −31.2 ± 4.3, −32.3 ± 1.3, and −30.7 ± 1.5 mV for wt MS2, T71K/G73R, and T71R/G73K, respectively (Figure 4D). The similarity of the zeta potentials is corroborated by the isoelectric points (pIs) of each variant and the p*K*_a_s of each residue between the single and double mutants calculated using PROPKA p*K*_a_ values (Figure S12, Tables S5 and S6).^36,37^ Negative zeta potential measurements for all capsid variants confirmed our prediction that locally supercharged MS2 particles would retain their negative charge overall. This differs from other most other well-characterized cationic protein delivery vehicles and supercharged proteins, which generally possess an overall net positive charge that can be visualized via theoretical electrostatic surface potential maps (Figure 4A).

**Figure 5:**
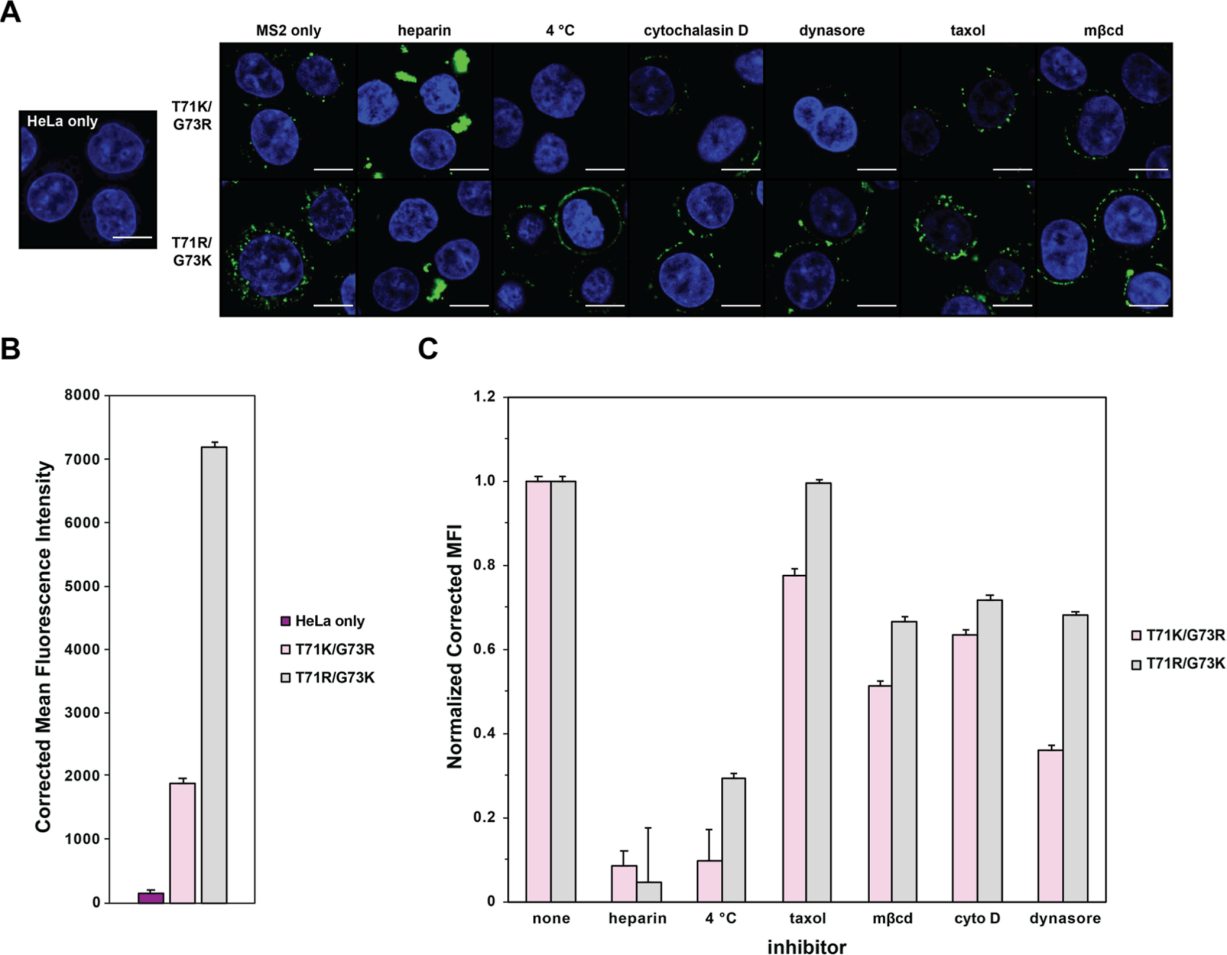
(A) Cellular uptake of MS2 T71K/G73R-fluorescein and MS2-T71R/G73K-fluorescein in the presence of endocytosis inhibitors assessed using confocal microscopy. Fixed cell images of HeLa cells treated with 5 μM MS2 T71K/G73R/N87C-fluorescein and the indicated endocytosis inhibitor. (B) Bar graphs showing corrected MFI of MS2-fluorescein internalization into HeLa cells. Error bars show the CV. Data shown are a concatenation of three biological replicates as measured by flow cytometry. (C) Bar graphs showing normalized cellular uptake of 5 μM MS2 T71K/G73R and MS2 T71R/G73K in the presence of endocytosis inhibitors. Data shown are a concatenation of three biological replicates as measured by flow cytometry. Error bars show the normalized CV.

To gain some preliminary insights into the mechanism by which MS2 capsids with positively charged surface residues enter cells, internalization experiments were repeated after treating cells with drugs that inhibit different endocytosis pathways and quantitated by flow cytometry.^38^ Treatment of cells with cytochalasin D, dynasore, Taxol, or methyl-β-cyclodextrin (MβCD) resulted in varied reduction of MS2 T71K/G73R or MS2 T71R/G73K internalization as measured by flow cytometry, but no major morphological changes in uptake were observed in confocal microscopy images (Figure 5). Each of these inhibitors targets a different mechanism of endocytosis, with dynasore preventing the bifurcation of clathrin-coated endosomes, Taxol stabilizing microtubules, and MβCD extracting cholesterol from the plasmid membrane.^38−40^ However, it is known that these inhibitors are not always direct and specific, and it is likely that MS2 T71K/G73R can enter cells through multiple endocytic pathways. Uptake of MS2 T71K/G73R and MS2 T71R/G73K was greatly reduced in cells cooled to 4 °C or treated with heparin, a heavily sulfated glycosaminoglycan, before and during MS2 exposure (Figure 5 and Table S4). These results suggest that uptake of charged MS2 variants is an energy-dependent process that may depend on initial capsid binding to cell surface heparan sulfate proteoglycans. This is consistent with previous reports of other positively charged molecules, such as HIV-Tat and +36 GFP, that require binding for internalization.^11,38,41^

To test for potential cytotoxic effects during internalization experiments, up to 20 μM concentrations (based on CP monomer) of either wtMS2 or the best-performing variants were incubated with HeLa and U-87 MG cells for 45 min (Figure S5C,D). Under normal treatment concentrations of 5 μM MS2, low toxicity was observed in both HeLa (<12%) and U-87 MG (<4%) cells. Toxicity increased in both cell types with higher MS2 concentrations, reaching 28% and 22% cytotoxicity for HeLa and U-87 MG cells at a treatment concentration of 20 μM MS2. Similar cytotoxicity levels were observed whether cells were treated with wtMS2, MS2 T71K/G73R, or MS2 T71R/G73K, suggesting that these uptake-enhanced variants do not result in the increased cytotoxicity common to globally cationic materials.^42,43^

A hemolysis assay was also performed to evaluate the potential hemolytic activity of the best-performing MS2 variants (Figure S6C).^44^ Up to 20 μM MS2 was incubated with red blood cells (RBCs) for 3 h at 37 °C, and released heme was measured. As a positive control, RBCs were also incubated with Lipofectamine 2000, a cationic lipid-based DNA transfection reagent that is known to cause hemolysis and other cytotoxic effects.^45,46^ Very little (1%) hemolysis was observed in MS2-treated samples, even at the highest concentration of 20 μM MS2. Increasing hemolysis was observed in the Lipofectamine 2000-treated cells, with up to 7.3 ± 1.4% hemolysis in RBCs treated with the highest Lipofectamine 2000 concentration of 0.05 mg/mL. Hemolysis levels did not change in cells treated with different MS2 variants. Taken together, these results indicate that capsids with positively charged surface residues have the potential to be safe and effective *in vivo* delivery vehicles for biotherapeutics.

### Cationic MS2-Mediated Delivery of MMAE to Glioblastoma Cells

**Figure 6:**
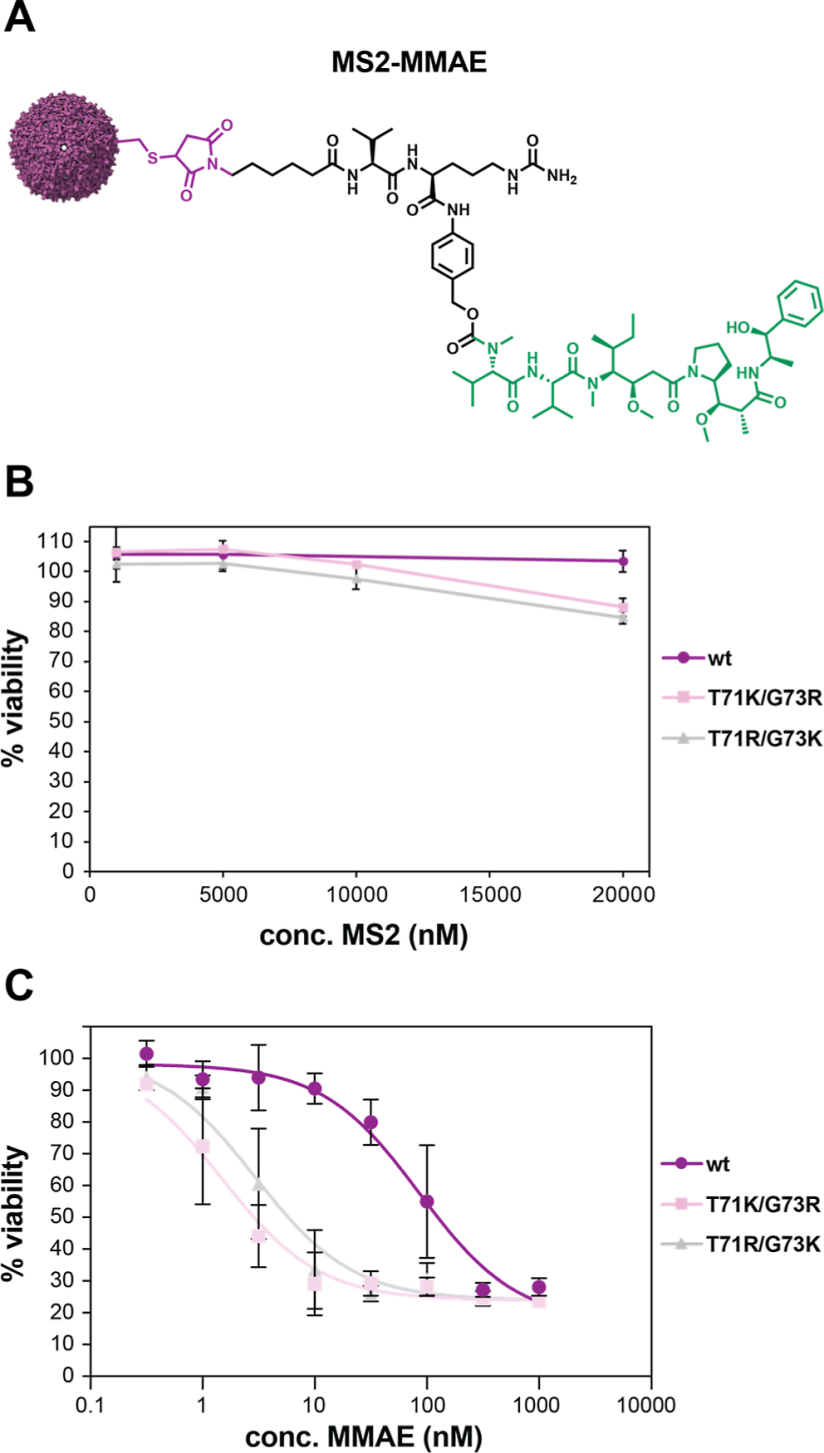
(A) Structure of the MS2-MMAE conjugate. MS2 capsid with interior cysteine conjugated to maleimide handle (purple), Val-Cit linker and spacer (black), and MMAE (green). (B) MTS viability assay of 1, 5, 10, and 20 μM wtMS2 (purple circles), MS2 T71K/G73R (pink squares), or MS2 T71R/G73K (gray triangles) incubated with U-87 MG cells for 72 h. Plot shows average % viability of three biological replicates. Error bars represent one standard deviation. (C) MS2-MMAE doseresponse assay. Results are shown for an MTS assay of U-87 MG cells 72 h after treatment with 0.5–1000 nM MMAE. Plot shows average % viability of three biological replicates. Error bars show one standard deviation.

We next determined whether the uptake-enhancing mutations would lead to enhanced efficacy for a drug molecule. MMAE is a potent antimitotic drug that is generally employed as a covalent construct to cancer-targeting monoclonal antibodies (mAbs) to produce antibody-drug conjugates (ADCs). Engineered mAbs with substituted cysteines, known as THIOMABs, have been developed to allow the conjugation of drug molecules to antibodies using maleimide handles.^47^ A dipeptide Val-Cit linker is typically incorporated as it is cleaved by the cathepsin B lysosomal protease upon cell entry to release the free drug in a targeted delivery approach.^48−50^ The half maximal inhibitory concentration (IC_50_) of MMAE has been measured to be close to 1 nM in multiple cancer cell lines in *in vitro* experiments, and similar inhibitory concentrations have been calculated for MMAE-ADCs as well.^51^ We hypothesized that conjugating MMAE to MS2 variants with positively charged surface residues could produce an equally or even more potent dose response given its potential to load up to 180 copies of a drug molecule per capsid, in comparison with only two drug molecules per mAb in most ADCs.^51^ Maleimide-Val-Cit-PAB-MMAE was conjugated to the interior cysteine of wtMS2, MS2 T71K/G73R, and MS2 T71R/G73K (Figure 6A). Each MS2 CP variant was modified to 92–97% by monomer with MMAE or about 165–175 molecules per capsid (Figure S11).

To test for potential cytotoxic effects during multi-day dose-response experiments, up to 20 μM drug-free MS2 was first incubated with U-87 MG cells for up to 72 h. Cell viability decreased slightly with higher MS2 concentrations, but cells treated with all three MS2 variants were >85% viable after 72 h (Figures 6B and S6A,B). The efficacy of MS2-MMAE was next measured by a dose-response assay in U-87 MG cells. Cells were treated for 72 h with up to 1000 nM MMAE conjugated to either wtMS2, MS2 T71K/G73R, or MS2 T71R/G73K. The calculated IC_50_ values for MMAE treatment with each MS2 variant were 85.30, 1.48, and 2.95 nM for wtMS2, MS2 T71K/G73R, and MS2 T71R/G73K, respectively (Figure 6C). These data show that MMAE delivery with engineered variants results in up to a 57-fold increase in efficacy versus delivery of MMAE with wtMS2, and a treatment concentration of only 10 pM assembled capsids is needed to elicit this potent cytotoxic response. Additionally, the IC_50_ for MS2-MMAE treatment with MS2 variants with positively charged surface residues is comparable to that of MMAE alone and MMAE-ADCs, demonstrating that locally supercharged MS2 capsids can be an effective delivery vehicle for potent anticancer therapeutics.^51^ It was perhaps unanticipated that both engineered capsids performed similarly in drug delivery, but there are several possible explanations, such as the different time and concentration scales for the fluorescently labeled MS2 uptake experiments (1 h, 5 μM CP monomers) versus MMAE delivery (72 h, 0.5 nM to 1μM CP monomers). We are in the process of confirming these differences further, but it remains true that both capsids have substantially better delivery performance in comparison with wtMS2.

## Conclusions

Previous work has established fitness landscaping as a successful method for identifying MS2 CP variants with improved properties such as increased thermostability, acid sensitivity, and chemical modifiability.^5,15,18^ Here, we capitalize on the nanoscale assembly of MS2-based VLPs to create locally supercharged variants with positively charged surface residues for increased mammalian cell uptake. Two key double mutants with positive charge concentrated at capsid pores were identified and found to exhibit greatly increased cellular penetration compared to wtMS2. Internalization of these variants was observed for multiple cell types, and the capsids were found to have low cytotoxic and hemolytic activity. These features add to their promise as effective drug delivery vehicles. When conjugated to MMAE, a potent anticancer small-molecule drug, MS2 T71K/G73R and MS2 T71R/G73K can deliver MMAE to glioblastoma cells and produce cytotoxic responses with low-nanomolar efficacies that are comparable to those of MMAE-ADCs. This demonstrates that MS2 variants with positively charged surface residues can be effective small-molecule delivery vehicles. These results show that this design concept can be applied to drug delivery, and further studies could help overcome current limits in delivery technology by optimizing the encapsulation and delivery of other small-molecule- and nucleic acid-based therapeutics. In current work, we are evaluating the *in vivo* characteristics of these promising delivery vehicles, as well as their ability to transfer other cargo types, such as proteins and nucleic acids, into cancer cells.

## Methods

### Safety Note

No unexpected or unusually high safety hazards were encountered. MMAE is a highly toxic drug and should be handled with care.

### Cloning Procedure

EMPIRIC cloning was used to generate all MS2 variants.^52^ Entry vector plasmids containing self-encoded removable fragments flanked by inverted BsaI recognition sites were previously used to generate all single amino acid mutations of the MS2 CP. Two single-stranded DNA primers were purchased, resuspended, and diluted to 50 ng/μL for each MS2 CP variant. Overlap extension PCR with the corresponding primer completed the reverse strand, and the double-stranded DNA was purified by Wizard SV Gel and PCR Clean-Up System, diluted to 1–5 ng/μL, and cloned into the corresponding entry vector using Golden Gate cloning. Ligated plasmids were digested with DpnI to remove methylated template DNA and transformed into chemical competent DH10B *Escherichia coli* cells. Cells were plated onto LB agar plates containing 20 μg/mL chloramphenicol and grown overnight at 37 °C. Individual colonies were picked and grown overnight in 2× YT media containing 20 μg/mL chloramphenicol at 37 °C, and then, plasmid DNA was isolated by the Zyppy plasmid miniprep kit and sent for sequencing.

### Expression and Purification of Proteins

Overnight cultures of each MS2 variant were subcultured 1:200 into 1L of 2× YT media containing 20 μg/mL chloramphenicol and grown to an OD_600_ of 0.4–0.6, and then induced with 0.1% w/v arabinose. Proteins were expressed overnight at 37 °C; then, cells were harvested by centrifugation and lysed by sonication. MS2 capsids were precipitated with one round of 50% ammonium sulfate (single positive charge mutants) or 20% PEG-6000 (double positive charge mutants). Large-scale expressions of well-assembled MS2 variants were purified via FPLC with a HiScreen Capto Core 700 column via isocratic flow with 10 mM phosphate buffer at pH 8.5 with 2 mM sodium azide as the equilibration, wash, and elution buffer, and 1 M NaOH in 30% isopropanol as the cleaning-in-place buffer.

### Determination of the Assembly State

The assembly state of each MS2 variant was characterized via HPLC size exclusion chromatography with an Agilent bioSEC-5 column (5 μm, 2000 Å, 7.8 × 300 mm) with an isocratic flow of 10 mM phosphate buffer at pH 7.2 with 2 mM sodium azide. Coelution of A_260_ and A_280_ peaks from 5 to 8 min is indicative of assembled capsids, while a peak at 10 min corresponds to the elution of unassembled MS2 dimers.

### Mass Spectrometry

Modified and unmodified MS2 CP variants were analyzed with an Agilent 1260 series liquid chromatograph connected in-line with an Agilent 6530 LC/QTOF mass spectrometer with an electrospray ionization source. The expected mass of each MS2 CP variant was confirmed via QTOF-ESI-MS (Figure S1).

### Dye Modification

All MS2 CP variants containing the N87C mutation (final concentration 10 μM) were mixed with 5 equiv of either fluorescein-5-maleimide (stock solution 5 mM in DMSO, 50 μM final concentration) in 10 mM phosphate buffer, pH 7.2. The solution was incubated on a rotator at room temperature for 1 h or at 4 °C overnight. Excess dye was removed by three rounds of washes through Amicon Ultra-0.5 mL 100 kDa MWCO filters and two rounds of filtration through Microspin G-25 columns. Modification of MS2 CP variants was verified via QTOF-ESI-MS and SDS-PAGE (Figures S2 and S3). Each MS2 CP variant was modified to 70–90% by monomer with dye, corresponding to 126–162 fluorophores per assembled capsid (Table S1). The concentration of each MS2-fluorophore conjugate was normalized to 5 μM for subsequent internalization assays.

### Synthesis of MMAE-Conjugated MS2 Capsids

Samples of each MS2 construct were prepared at 20 μM and combined with 10 equiv of a stock solution of MC-Val-Cit-PAB-MMAE (BroadPharm) in 100 mM phosphate buffer (1% v/v DMSO) pH 7.4 and incubated overnight at 4 °C. Excess MMAE was removed via seven cycles of spin filtration through a 100 kDa MWCO filter (Amicon), and product was analyzed by QTOF-ESI-MS.

### Hemolysis Assay

A hemolysis assay was performed to measure potential damage to RBCs by quantifying the release of iron-containing hemoglobin into plasma after incubation with different MS2 CP variants.^53^ A Hemoglobin Assay Kit was used according to Sigma-Aldrich’s instructions to quantify the release of heme from human whole blood after incubation with different MS2 CP variants. Briefly, this assay uses the Triton/NaOH method in which hemoglobin is converted to a colored product that can be measured by absorbance at 400 nm. Single donor human whole blood containing anti-coagulant was obtained from VWR and stored at 4 °C for up to 48 h. A 1 mL aliquot of blood was centrifuged for 15 min at 800*g*. The supernatant was collected and used to determine the amount of plasma-free hemoglobin. Standard curves of whole blood and plasma were prepared to determine the optimal concentration of blood for total blood hemoglobin and plasma blood hemoglobin measurements, respectively. The remaining whole blood was spun down for 10 m at 500*g* and resuspended in 1× PBS (Ca^2+^/Mg^2+^-free) to remove any anti-coagulant. This wash step was repeated twice, and the final pellet was diluted in 1× PBS (Ca^2+^/Mg^2+^-free) to adjust total hemoglobin concentration to 10 mg/mL. Tubes were prepared with 100 μL of the diluted whole blood, 800 μL of Ca^2+^/Mg^2+^-free PBS, and 100 μL of MS2 variant stocks to give final treatment concentrations of 0, 0.1, 1, 5, 10, and 20 μM MS2 in a total volume of 1 mL. For comparison with another delivery agent, tubes were also prepared with 100 μL of the diluted whole blood, 800 μL of Ca^2+^/Mg^2+^-free PBS, and 100 μL of Lipofectamine 2000 stocks to give final treatment concentrations of 0.003, 0.006, 0.013, 0.025, and 0.050 mg/mL Lipofectamine 2000 in a total volume of 1 mL. The Lipofectamine treatment concentrations were chosen to correspond to the suggested reagent concentration range according to the Thermo Fisher user protocol. A maximum lysis treatment was also prepared with 100 μL of diluted whole blood, 800 μL of Ca^2+^/Mg^2+^-free PBS, and 100 μL of 10× CyQUANT Cell Lysis Buffer. The tubes were placed in a 37 °C water bath for 3 h and mixed gently every 30 min. The samples were then centrifuged for 15 min at 800*g*. The supernatant was collected, the hemoglobin concentration was measured according to the kit instructions, and percent hemolysis was calculated according to the following equation
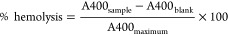


### Lactate Dehydrogenase Cytotoxicity Assay

A CyQUANT Lactate Dehydrogenase (LDH) Cytotoxicity Assay Kit was used according to Thermo Fisher’s instructions to quantify possible cytotoxicity mediated by different MS2 CP variants. Briefly, LDH is released into cell culture media by damage to the plasma membrane. Extracellular LDH can be measured by an enzymatic reaction where LDH catalyzes the conversion of lactate to pyruvate via NAD+ reduction to NADH coupled to a colorimetric readout at 490 nm. The optimum HeLa and U-87 MG cell numbers for the LDH assay were determined by plating two sets of serial dilutions of 0–10,000 cells/100 μL media in a 96-well plate. After overnight incubation, 10 μL of sterile water was added to each well of one cell dilution series for the spontaneous LDH release control, and 10 μL of 10× lysis buffer was added to the other cell dilution series for the maximum LDH release control and incubated at 37 °C with 5% CO_2_ for 45 min. LDH activity was measured according to the kit instructions, and the linear range of the assay for HeLa and U-87 MG cells was determined (Figure S4A,B). The optimal number of cells (4000/well) was plated with 100 μL of media in triplicate wells in a 96-well plate and incubated overnight at 37 °C with 5% CO_2_. A combination of 10 μL of sterile water, 10 μL of lysis buffer, and 10 μL of 1, 5, 10, or 20 μM MS2 was each added to one set of triplicate wells. The resulting samples were incubated at 37 °C with 5% CO_2_ for 45 min. LDH activity was measured according to the kit instructions, and the percent cytotoxicity for each MS2 treatment concentration was calculated using the following formula:
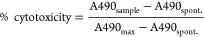


### MTS Cell Viability Assay

An MTS Assay Kit (ab197010) was used according to Abcam’s instructions to quantify cell viability after treatment with different MS2 CP variants. Briefly, an MTS tetrazolium compound is reduced by viable mammalian cells to generate a colored formazan dye that can be measured by absorbance at 490 nm. HeLa and U-87 MG cells were plated into a 96-well plate at a concentration of 5000 cells per well in 100 μL of DMEM + fetal bovine serum (FBS) and incubated overnight at 37 °C with 5% CO_2_. The next day, the medium was aspirated and replaced with 100 μL of either 0, 1, 5, 10, 20, or 40 μM MS2 diluted in DMEM + FBS and incubated for 24, 48, or 72 h at 37 °C with 5% CO_2_. Then, the treatment medium was aspirated, and 100 μL of DMEM + 20% MTS reagent was added to each well and incubated for 1 h at 37 °C with 5% CO_2_ before measuring the absorbance at 490 nm. Cell viability was calculated as an absorbance percentage over the untreated control:
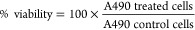


### MS2-MMAE MTS Cell Viability Assay

U-87 MG cells were plated in a 96-well plate at 4000 cells/well and allowed to adhere overnight. Cells were then incubated for 72 h at 37 °C with 80 μL of DMEM + 10% FBS and 20 μL of MS2-MMAE or vehicle control at appropriate concentrations in DPBS. Media were then aspirated and replaced with 200 μL of pre-warmed DMEM without phenol red or FBS along with 20 μL of Abcam MTS cell proliferation assay solution. Samples were then incubated in the dark for 1 h and immediately analyzed for optical density at 490 nm. Cell viability was calculated as an absorbance percentage over the untreated control:
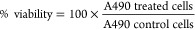


### Mammalian Cell Culture

HeLa, Saos-2, HEK293T, and U-87 MG cells were cultured in DMEM supplemented with 10% FBS, 4.5 g/L glucose, 4 mM l-glutamine, and 1 mM sodium pyruvate. All cell cultures were maintained at 37 °C in a humidified atmosphere with 5% CO_2_.

### Epifluorescence Microscopy

25,000 HeLa cells were plated in each well of a 96-well plate and incubated overnight at 37 °C with 5% CO_2_. Cells were washed three times with DPBS and treated with 5 μM MS2-fluorescein in DPBS with 1% FBS for 1 h at 37 °C with 5% CO_2_. After incubation, cells were washed three times with 20 U/mL heparin in DPBS to remove positively charged surface-bound species. Cells were fixed with 2% paraformaldehyde and 0.1 μg/mL DAPI and kept at 4 °C until imaging. Cells were imaged on an ImageXpress Micro fluorescent microscope.

### Flow Cytometry

200,000 HeLa cells were plated in each well of a 24-well plate and incubated overnight at 37 °C with 5% CO_2_. Cells were washed three times with DPBS and treated with 5 μM MS2-fluorescein in DPBS with 1% FBS for 1 h at 37 °C with 5% CO_2_. After incubation, cells were washed three times with 20 U/mL heparin in DPBS to remove positively charged surface-bound species. Cells were lifted with trypsin, quenched with FBS-containing DMEM, and pelleted at 200*g* for 3 min. Cells were washed twice with DPBS and resuspended in 1 mL of DPBS. Cells were pelleted at 200*g* for 3 min, resuspended in 1 mL of 2% paraformaldehyde with 0.1 μg/mL DAPI, and kept at 4 °C until flow cytometry. Flow cytometry was completed using an Attune NxT flow cytometer. At least 10,000 cells were analyzed for each sample. Data were analyzed using FlowJo, and the mean fluorescence values with CV were reported (Table S2, Figures S6 and S7A). Fluorescence intensity values were corrected according to the % fluorescein-maleimide modification of each MS2 CP variant (Table S2 and Figure S6).

### Flow Cytometry for Multiple Cell Type Screen

HeLa, Saos-2, HEK293T, and U-87 MG cells were plated at 60% confluence in each well of a 12-well plate and incubated overnight 37 °C with 5% CO_2_. Cells were washed three times with DPBS and treated with 5 μM MS2-fluorescein in DPBS with 1% FBS for 1 h at 37 °C with 5% CO_2_. Cells were lifted with trypsin, quenched with FBS-containing DMEM, and pelleted at 200*g* for 3 min. Cells were washed twice with DPBS and resuspended in 1 mL of DPBS. Cells were pelleted at 200*g* for 3 min, resuspended in 1 mL 2% paraformaldehyde with 0.1 μg/mL DAPI, and kept at 4 °C until flow cytometry. Flow cytometry was completed using an Attune NxT flow cytometer. At least 10,000 cells were analyzed for each sample. Data were analyzed using FlowJo, and the mean fluorescence values with CV were reported (Table S3 and Figure S7C).

### Endocytosis Inhibitor Screen

50,000 cells/well were plated in fibronectin-coated 8-well Nunc Lab-Tek I Chambered Coverglass slides, and 200,000 cells/well were plated in 24-well plates and incubated overnight at 37 °C with 5% CO_2_. Cells were washed three times with DPBS and treated with either 10 U/mL heparin, 5 μM cytochalasin D, 80 μM dynasore, 20 μM Taxol, or 5 mM MβCD in DPBS with 1% FBS for 30 min at 37 °C with 5% CO_2_. Separate plates of cells were incubated with DPBS with 1% FBS at 4 °C for 30 min. Cells were washed with DPBS and treated with 5 μM MS2-fluorescein in DPBS with 1% FBS for 1 h at 37 °C with 5% CO_2_ and either 10 U/mL heparin, 5 μM cytochalasin D, 80 μM dynasore, 20 μM Taxol, or 5 mM MβCD. Separate plates of cells were incubated with 5 μM MS2-fluorescein in DPBS with 1% FBS at 4 °C for 1 h. After incubation, cells were washed three times with 20 U/mL heparin in DPBS to remove positively charged surface-bound species. Cells in the 8-well microscopy slides were fixed with 2% paraformaldehyde and 0.1 μg/mL DAPI and kept at 4 °C until imaging. Cells were imaged on a Zeiss LSM 880 confocal microscope. Cells in the 24-well plates were lifted with trypsin, quenched with FBS-containing DMEM, and pelleted at 200*g* for 3 min. Cells were washed twice with DPBS, resuspended in 1 mL of 2% paraformaldehyde with 0.1 μg/mL DAPI, and kept at 4 °C until flow cytometry. Flow cytometry was completed using an Attune NxT flow cytometer. At least 10,000 cells were analyzed for each sample. Data were analyzed using FlowJo, and the median fluorescence values with CV were reported (Table S4 and Figure S7B).

### Molecular Mechanics Calculations

An assembly of 12 wtMS2 CP dimers (i.e., 24 MS2 monomers in total) was generated around the threefold axis of symmetry (PDB ID: 2MS2). An assembly of 10 wtMS2 dimers (20 monomer proteins in total) surrounding the fivefold axis of symmetry was prepared in the same manner. The PyMOL mutagenesis wizard tool was used to create all MS2 CP variants. The structures were imported into Schrodinger’s Maestro suite (version 2021-2), and the structures were preprocessed with Maestro’s protein preparation wizard. Hydrogen bonds were assigned with the H-bond optimization tool at a PROPKA pH of 7. Then, a restrained minimization of the structure using the OPLS4 forcefield was performed. MacroModel was used to run a large-scale low mode conformational search of the minimized structures. All residues within 10 Å of the mutations were allowed to move freely. All residues between 10 and 20 Å of the mutations were restrained with a force constant of 200 kJ/mol. All atoms beyond these subshells were frozen in place. Sampling used 1000 maximum steps with 100 steps per 100 rotatable bond. The top five output structures for each variant were compared to confirm similar conformations for the mutated residues.

### pI and p*K*_a_ Calculations

The sequence-based pIs of wt MS2, MS2 T71K/G73R, and MS2 T71R/G73K were calculated using the ExPaSy Compute pI/MW tool and the pI calculator 2.0 tool (Table S5).^54,55^ Structure-based pIs of wt MS2, MS2 T71K/G73R, and MS2 T71R/G73K were calculated from the output structures of the described MacroModel molecular mechanics simulations. The protein titration curve tool in Maestro was used to determine the pI of the 10mer and 12mer structures of each variant and the PROPKA p*K*_a_s of each residue at positions 71 and 73 (Table S6 and Figure S12).^37^

### DLS Size Measurements

MS2 samples were prepared by diluting to 50 μM in 10 mM sodium phosphate, pH 7.2, and passing through a 0.2 μM centrifugal filter. The filtered samples were added to a DLS cuvette, and 25 °C was used for room-temperature analysis. Size measurements were taken in triplicate sets of 13 runs each. The diameter and standard deviation for each MS2 CP variant were calculated from an average of the % volume mean from each run.

### DLS Temperature-Dependent Size Measurements

MS2 samples were prepared by diluting to 50 μM in 10 mM sodium phosphate, pH 7.2, and passing through a 0.2 μM centrifugal filter. The filtered samples were added to a DLS cuvette, and measurements were taken with increasing temperature from 25 to 75 °C at 5 °C intervals. The diameter and standard deviation for each MS2 CP variant were calculated from an average of the % volume mean from each run.

### DLS Zeta Potential Measurements

MS2 samples were prepared by diluting to 50 μM in 10 mM sodium phosphate, pH 7.2, and passing through a 0.2 μM centrifugal filter. The filtered samples were added to a Malvern DTS1070 Folded Capillary Cell, and 25 °C was used for room-temperature analysis. Zeta potential measurements were taken in triplicate sets of 100 runs each. The zeta potential average and standard deviation for each MS2 CP variant were reported in millivolts.

## Supporting Information Available

Materials and equipment information, DNA primer sequences, sequences of expressed proteins, supplementary figures and tables, MS2 capsid assembly confirmation, intact mass spectra of unmodified and modified MS2 CP variants, supplementary cytotoxicity assay data, supplementary flow cytometry and microscopy data, electrostatic surface potential maps of MS2 CP variants, expected masses of unmodified and modified MS2 CP variants, and flow cytometry statistical data (PDF)

## Abbreviations

VLP: virus-like particle
NP: nanoparticle
SyMAPS: Systematic Mutation and Assembled Particle Selection
CP: coat protein
AFL: apparent fitness landscape
AFS: apparent fitness score
MFI: mean fluorescence intensity
DLS: dynamic light scattering
pI: isoelectric point
RBC: red blood cell
MMAE: monomethyl auristatin E
mAb: monoclonal antibody
wt: wild-type
