# Supplementary material for: Fitness Landscape-Guided Engineering of Locally Supercharged Virus-like Particles with Enhanced Cell Uptake Properties
Source: ACS Chem Biol. 2022 Nov 15;17(12):3367–78. doi: 10.1021/acschembio.2c00318 (PMC9764284; doi:10.1021/acschembio.2c00318)
Supplement: Supplementary file 1 — cb2c00318_si_001.pdf [file cb2c00318_si_001.pdf]

Fitness landscape-guided engineering of locally supercharged virus-like particles with enhanced cell uptake properties

Paige E. Pistono[a], Paul Huang[a], Daniel D. Brauer[a], & Matthew B. Francis[ab*]

[a]Department of Chemistry, University of California, Berkeley, CA 94720, United States

[b]Materials Sciences Division, Lawrence Berkeley National Laboratory, Berkeley, CA 94720, United States

*Corresponding author. Email: mbfrancis@berkeley.edu

SUPPORTING INFORMATION

Table of Contents

Materials **2**

Equipment **2**

DNA Primer Sequences **3**

Sequences of Expressed Proteins **4**

Supplementary Figures **6**

Supplementary Tables **21**

References **26**

Materials

General Information. All reagents were obtained from commercial sources and used without further purification unless otherwise indicated. Milli-Q (MQ) H2O was purified to a resistivity of 18.2 M Ω•cm (at 25 °C) using a MQ Gradients ultrapure water purification system from Millipore (Burlington, MA).

Plasmid design, protein expression and purification. DNA primers for Golden Gate Assembly were purchased from Integrated DNA Technologies (Coralville, IA). Phusion™ High-Fidelity DNA Polymerase, Phusion™ HF Buffer, deoxynucleotide (dNTP) solution mix, T4 DNA ligase, BsaI and DpnI were purchased from New England BioLabs (Ipswich, MA). Wizard® SV Gel and PCR Clean-Up System was purchased from Promega (Madison, WI). *E. coli* DH10B cells were purchased from the UC Berkeley MacroLab (Berkeley, CA). Zyppy™ plasmid miniprep kit was purchased from Zymo Research (Irvine, CA). DNA sequencing was performed by Sequetech. Corning® Costar® Spin-X® centrifuge tube filters, Amicon Ultra-0.5 mL 100 kDa MWCO filters, and 2x YT medium powder were purchased from Sigma Aldrich (St. Louis, MO). HiScreen Capto Core 700 column, Microspin™ G-25 columns, and Amersham NAP-5 columns were purchased from Cytiva Lifesciences (Marlborough, MA). Bio SEC-5 HPLC column was purchased from Agilent (Santa Clara, CA). NuPAGE™ 4 to 12%, Bis-Tris precast gels, NuPAGE™ MES SDS Running Buffer and Fluorescein-5-Maleimide were purchased from Thermo Fisher (Waltham, MA).

Cell culture and assays. HeLa, U-87 MG, Saos-2, and HEK293T cells were purchased from the UC Berkeley Cell Culture Facility. Dulbecco's modified eagle medium (DMEM, with and without Phenol Red), Dulbecco's phosphate-buffered saline (DPBS), TrypLE, Hoechst 33342 stain, Invitrogen™ CyQUANT™ LDH Cytotoxicity Assay kit, glutamine, and sodium pyruvate were purchased from Thermo Fisher (Waltham, MA). MTS Assay Cell Proliferation Kit was purchased from Abcam (Cambridge, UK). Fibronectin was purchased from MilliporeSigma (Burlington, MA). Heparin sodium was purchased from Neta Scientific (Hainesport, NJ). Cytochalasin D, dynasore hydrate, taxol, and methyl-β-cyclodextrin were purchased from Sigma Aldrich (St. Louis, MO).

Equipment

For plasmid and protein concentration quantification, a ND-1000 (NanoDrop) spectrophotometer was used. A Sorvall X Pro Series centrifuge was used for protein expression (Thermo Fisher). An AKTA start FPLC (Cytiva), with a HiScreen Capto Core 700 column was used for protein purification. A 1260 Infinity HPLC (Agilent) was used for MS2 assembly confirmation and a 6530 LC/QTOF (Agilent) mass spectrometer was used for characterization of MS2 variants. For cellular internalization, confocal microscopy, and fluorescence correlation spectroscopy, an Attune NxT Flow Cytometer (Thermo Fisher), Image Xpress Micro (Molecular Devices) and LSM 880 (Zeiss) were used. Dynamic light scattering was performed on a Zetasizer Nano ZS (Malvern Instruments). Absorption readings after MTS cell proliferation assays were performed on an Infinite 200 PRO plate reader (Tecan).

DNA Primer Sequences

Primer Name	Sequence (5' to 3')
EV3 G73R fwd	AGG TCT CAC TCT GCG CAG AAT CGC AAA TAC ACC ATC AAA GTC GAG GTG CCT AAA GTG GCA
EV3 G73R rev	AGG TCT CAC TAC AGG AAG CTC TAC ACC ACG AAC AGT CTG GGT TGC CAC TTT AGG CAC CTC
EV1 N12K fwd	AGG TCT CAC ATG GCT TCT AAC TTT ACT CAG TTC GTT CTC GTC GAC AAG GGC GGA ACT GGC
EV1 rev	AGG TCT CAC GTT AGC GAA GTT GCT TGG GGC GAC AGT CAC GTC GCC AGT TCC GCC ATT GTC
EV1 Q6R fwd	AGG TCT CAC ATG GCT TCT AAC TTT ACT CGG TTC GTT CTC GTC GAC AAT GGC GGA ACT GGC
EV1 T5H fwd	AGG TCT CAC ATG GCT TCT AAC TTT CAT CAG TTC GTT CTC GTC GAC AAT GGC GGA ACT GGC
EV1 T15H fwd	AGG TCT CAC ATG GCT TCT AAC TTT ACT CAG TTC GTT CTC GTC GAC AAT GGC GGA CAT GGC
EV1 T15H rev	AGG TCT CAC GTT AGC GAA GTT GCT TGG GGC GAC AGT CAC GTC GCC ATG TCC GCC ATT GTC
EV3 T71K fwd	AGG TCT CAC TCT GCG CAG AAT CGC AAA TAC ACC ATC AAA GTC GAG GTG CCT AAA GTG GCA
EV3 T71K rev	AGG TCT CAC TAC AGG AAG CTC TAC ACC ACC AAC TTT CTG GGT TGC CAC TTT AGG CAC CTC
EV3 T71K G73R fwd	AGG TCT CAC TCT GCG CAG AAT CGC AAA TAC ACC ATC AAA GTC GAG GTG CCT AAA GTG GCA
EV3 T71K G73R rev	AGG TCT CAC TAC AGG AAG CTC TAC ACC ACG AAC TTT CTG GGT TGC CAC TTT AGG CAC CTC
EV3 T71K V67R fwd	AGG TCT CAC TCT GCG CAG AAT CGC AAA TAC ACC ATC AAA GTC GAG GTG CCT AAA CGG GCA
EV3 T71K V67R rev	AGG TCT CAC TAC AGG AAG CTC TAC ACC ACC AAC TTT CTG GGT TGC CCG TTT AGG CAC CTC
EV3 V67R fwd	AGG TCT CAC TCT GCG CAG AAT CGC AAA TAC ACC ATC AAA GTC GAG GTG CCT AAA CGG GCA
EV3 V67R rev	AGG TCT CAC TAC AGG AAG CTC TAC ACC ACC AAC AGT CTG GGT TGC CCG TTT AGG CAC CTC
EV3 V67R G73R fwd	AGG TCT CAC TCT GCG CAG AAT CGC AAA TAC ACC ATC AAA GTC GAG GTG CCT AAA CGG GCA
EV3 V67R G73R rev	AGG TCT CAC TAC AGG AAG CTC TAC ACC ACG AAC AGT CTG GGT TGC CCG TTT AGG CAC CTC
EV3 V67R T71K G73R fwd	AGG TCT CAC TCT GCG CAG AAT CGC AAA TAC ACC ATC AAA GTC GAG GTG CCT AAA CGG GCA
EV3 V67R T71K G73R rev	AGG TCT CAC TAC AGG AAG CTC TAC ACC ACG AAC TTT CTG GGT TGC CCG TTT AGG CAC CTC
EV3 T71R fwd	AGG TCT CAC TCT GCG CAG AAT CGC AAA TAC ACC ATC AAA GTC GAG GTG CCT AAA GTG GCA
EV3 T71R rev	AGG TCT CAC TAC AGG AAG CTC TAC ACC ACC AAC TCT CTG GGT TGC CAC TTT AGG CAC CTC
EV3 G73K fwd	AGG TCT CAC TCT GCG CAG AAT CGC AAA TAC ACC ATC AAA GTC GAG GTG CCT AAA GTG GCA
EV3 G73K rev	AGG TCT CAC TAC AGG AAG CTC TAC ACC TTT AAC AGT CTG GGT TGC CAC TTT AGG CAC CTC
EV3 T71K G73K fwd	AGG TCT CAC TCT GCG CAG AAT CGC AAA TAC ACC ATC AAA GTC GAG GTG CCT AAA GTG GCA
EV3 T71K G73K rev	AGG TCT CAC TAC AGG AAG CTC TAC ACC TTT AAC TTT CTG GGT TGC CAC TTT AGG CAC CTC
EV3 T71R G73R fwd	AGG TCT CAC TCT GCG CAG AAT CGC AAA TAC ACC ATC AAA GTC GAG GTG CCT AAA GTG GCA
EV3 T71R G73R rev	AGG TCT CAC TAC AGG AAG CTC TAC ACC TCT AAC TCT CTG GGT TGC CAC TTT AGG CAC CTC
EV3 T71R G73K fwd	AGG TCT CAC TCT GCG CAG AAT CGC AAA TAC ACC ATC AAA GTC GAG GTG CCT AAA GTG GCA
EV3 T71R G73K rev	AGG TCT CAC TAC AGG AAG CTC TAC ACC TTT AAC TCT CTG GGT TGC CAC TTT AGG CAC CTC

Sequences of Expressed Proteins

MS2 wt
ASNFTQFVLVDNGGTGDVTVAPSNFANGVAEWISSNSRSQAYKVTCSVRQSSAQNRKYTIKVEVPKVATQT
VGGVELPVAAWRSYLNMELTIPIFATNSDCELIVKAMQGLLKDGNPIPSAIAANSGIY

MS2 N87C
ASNFTQFVLVDNGGTGDVTVAPSNFANGVAEWISSNSRSQAYKVTCSVRQSSAQNRKYTIKVEVPKVATQT
VGGVELPVAAWRSYLCMELTIPIFATNSDCELIVKAMQGLLKDGNPIPSAIAANSGIY

MS2 T5H/N87C
ASNFHQFVLVDNGGTGDVTVAPSNFANGVAEWISSNSRSQAYKVTCSVRQSSAQNRKYTIKVEVPKVATQT
VGGVELPVAAWRSYLCMELTIPIFATNSDCELIVKAMQGLLKDGNPIPSAIAANSGIY

MS2 Q6R/N87C
ASNFTRFVLVDNGGTGDVTVAPSNFANGVAEWISSNSRSQAYKVTCSVRQSSAQNRKYTIKVEVPKVATQT
VGGVELPVAAWRSYLCMELTIPIFATNSDCELIVKAMQGLLKDGNPIPSAIAANSGIY

MS2 N12K/N87C
ASNFTQFVLVDKGGTGDVTVAPSNFANGVAEWISSNSRSQAYKVTCSVRQSSAQNRKYTIKVEVPKVATQT
VGGVELPVAAWRSYLCMELTIPIFATNSDCELIVKAMQGLLKDGNPIPSAIAANSGIY

MS2 T15H/N87C
ASNFTQFVLVDNGGHGDVTVAPSNFANGVAEWISSNSRSQAYKVTCSVRQSSAQNRKYTIKVEVPKVATQT
VGGVELPVAAWRSYLCMELTIPIFATNSDCELIVKAMQGLLKDGNPIPSAIAANSGIY

MS2 V67R/N87C
ASNFTQFVLVDNGGTGDVTVAPSNFANGVAEWISSNSRSQAYKVTCSVRQSSAQNRKYTIKVEVPKRATQT
VGGVELPVAAWRSYLCMELTIPIFATNSDCELIVKAMQGLLKDGNPIPSAIAANSGIY

MS2 T71K/N87C
ASNFTQFVLVDNGGTGDVTVAPSNFANGVAEWISSNSRSQAYKVTCSVRQSSAQNRKYTIKVEVPKVATQK
VGGVELPVAAWRSYLCMELTIPIFATNSDCELIVKAMQGLLKDGNPIPSAIAANSGIY

MS2 G73R/N87C
ASNFTQFVLVDNGGTGDVTVAPSNFANGVAEWISSNSRSQAYKVTCSVRQSSAQNRKYTIKVEVPKVATQT
VRGVELPVAAWRSYLCMELTIPIFATNSDCELIVKAMQGLLKDGNPIPSAIAANSGIY

MS2 V67R/T71K/N87C
ASNFTQFVLVDNGGTGDVTVAPSNFANGVAEWISSNSRSQAYKVTCSVRQSSAQNRKYTIKVEVPKRATQK
VGGVELPVAAWRSYLCMELTIPIFATNSDCELIVKAMQGLLKDGNPIPSAIAANSGIY

MS2 V67R/G73R/N87C
ASNFTQFVLVDNGGTGDVTVAPSNFANGVAEWISSNSRSQAYKVTCSVRQSSAQNRKYTIKVEVPKRATQT
VRGVELPVAAWRSYLCMELTIPIFATNSDCELIVKAMQGLLKDGNPIPSAIAANSGIY

MS2 T71K/G73R/N87C
ASNFTQFVLVDNGGTGDVTVAPSNFANGVAEWISSNSRSQAYKVTCSVRQSSAQNRKYTIKVEVPKVATQK
VRGVELPVAAWRSYLCMELTIPIFATNSDCELIVKAMQGLLKDGNPIPSAIAANSGIY

MS2 V67R/T71K/G73R/N87C
ASNFTQFVLVDNGGTGDVTVAPSNFANGVAEWISSNSRSQAYKVTCSVRQSSAQNRKYTIKVEVPKRATQK
VRGVELPVAAWRSYLCMELTIPIFATNSDCELIVKAMQGLLKDGNPIPSAIAANSGIY

MS2 T71R/N87C
ASNFTQFVLVDNGGTGDVTVAPSNFANGVAEWISSNSRSQAYKVTCSVRQSSAQNRKYTIKVEVPKVATQR
VGGVELPVAAWRSYLCMELTIPIFATNSDCELIVKAMQGLLKDGNPIPSAIAANSGIY

MS2 G73K/N87C
ASNFTQFVLVDNGGTGDVTVAPSNFANGVAEWISSNSRSQAYKVTCSVRQSSAQNRKYTIKVEVPKVATQT
VKGVELPVAAWRSYLCMELTIPIFATNSDCELIVKAMQGLLKDGNPIPSAIAANSGIY

MS2 T71R/G73R/N87C
ASNFTQFVLVDNGGTGDVTVAPSNFANGVAEWISSNSRSQAYKVTCSVRQSSAQNRKYTIKVEVPKVATQR
VRGVELPVAAWRSYLCMELTIPIFATNSDCELIVKAMQGLLKDGNPIPSAIAANSGIY

MS2 T71K/G73K/N87C
ASNFTQFVLVDNGGTGDVTVAPSNFANGVAEWISSNSRSQAYKVTCSVRQSSAQNRKYTIKVEVPKVATQK
VKGVELPVAAWRSYLCMELTIPIFATNSDCELIVKAMQGLLKDGNPIPSAIAANSGIY

MS2 T71R/G73K/N87C
ASNFTQFVLVDNGGTGDVTVAPSNFANGVAEWISSNSRSQAYKVTCSVRQSSAQNRKYTIKVEVPKVATQR
VKGVELPVAAWRSYLCMELTIPIFATNSDCELIVKAMQGLLKDGNPIPSAIAANSGIY

Supplementary Figures

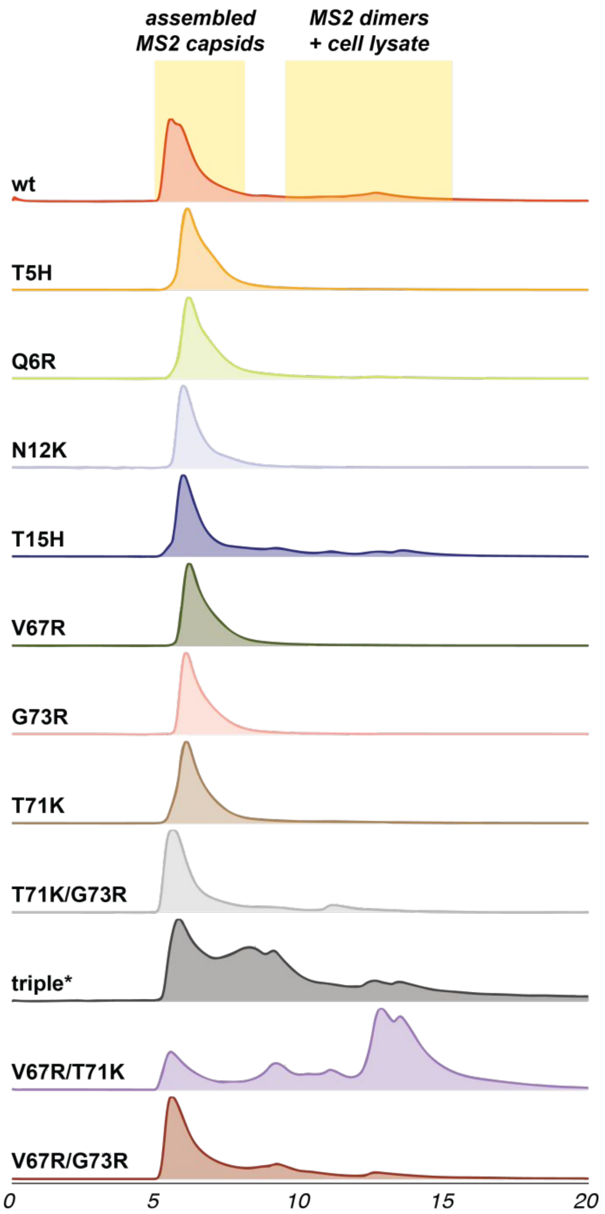

Figure S1. HPLC SEC traces of MS2 CP variants. Highlighted in yellow on the N87C trace are the expected elution times of assembled capsids (5-8 min) and dimers and cell lysate (10-15 min). *Triple corresponds to MS2 CP variant V67R/T71K/G73R.

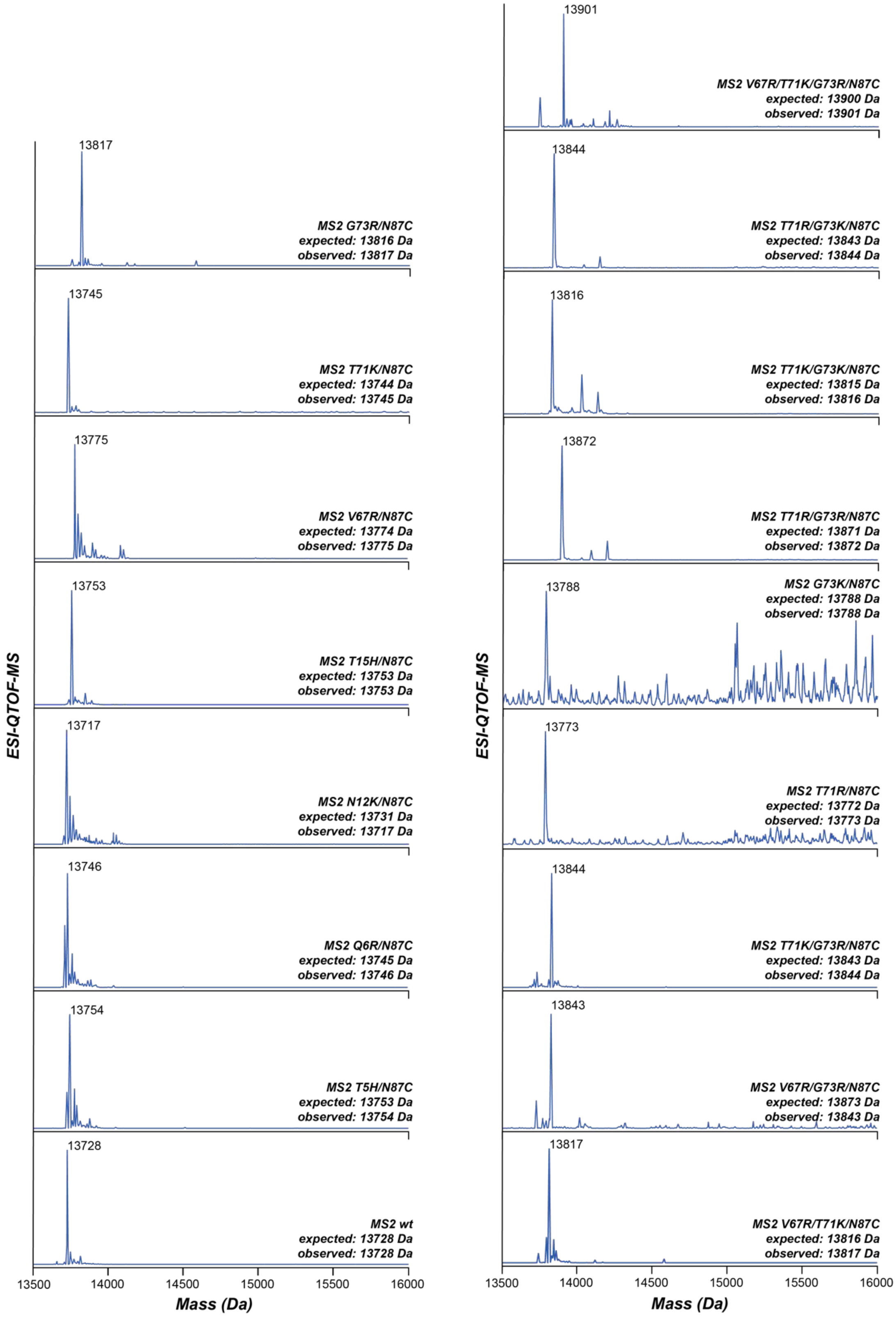

Figure S2. LC-MS analysis of MS2 CP variants.

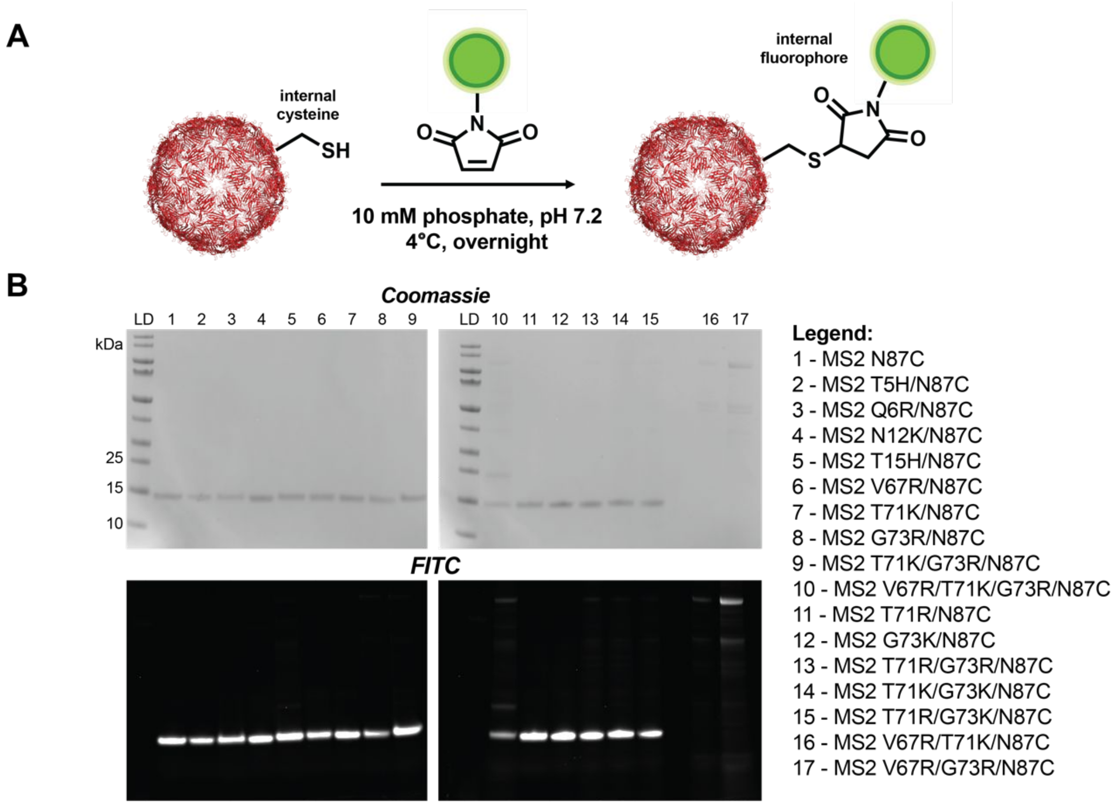

Figure S3. (A) Reaction scheme describing fluorophore-maleimide coupling to the internal cysteine N87C of each MS2 CP variant. **(B)** SDS-PAGE analysis illustrating the purity and normalization of each MS2-fluorescein conjugate before cell internalization assays. Each conjugate was normalized to 5 µM after fluorophore coupling and 15 µL was analyzed via SDS-PAGE.

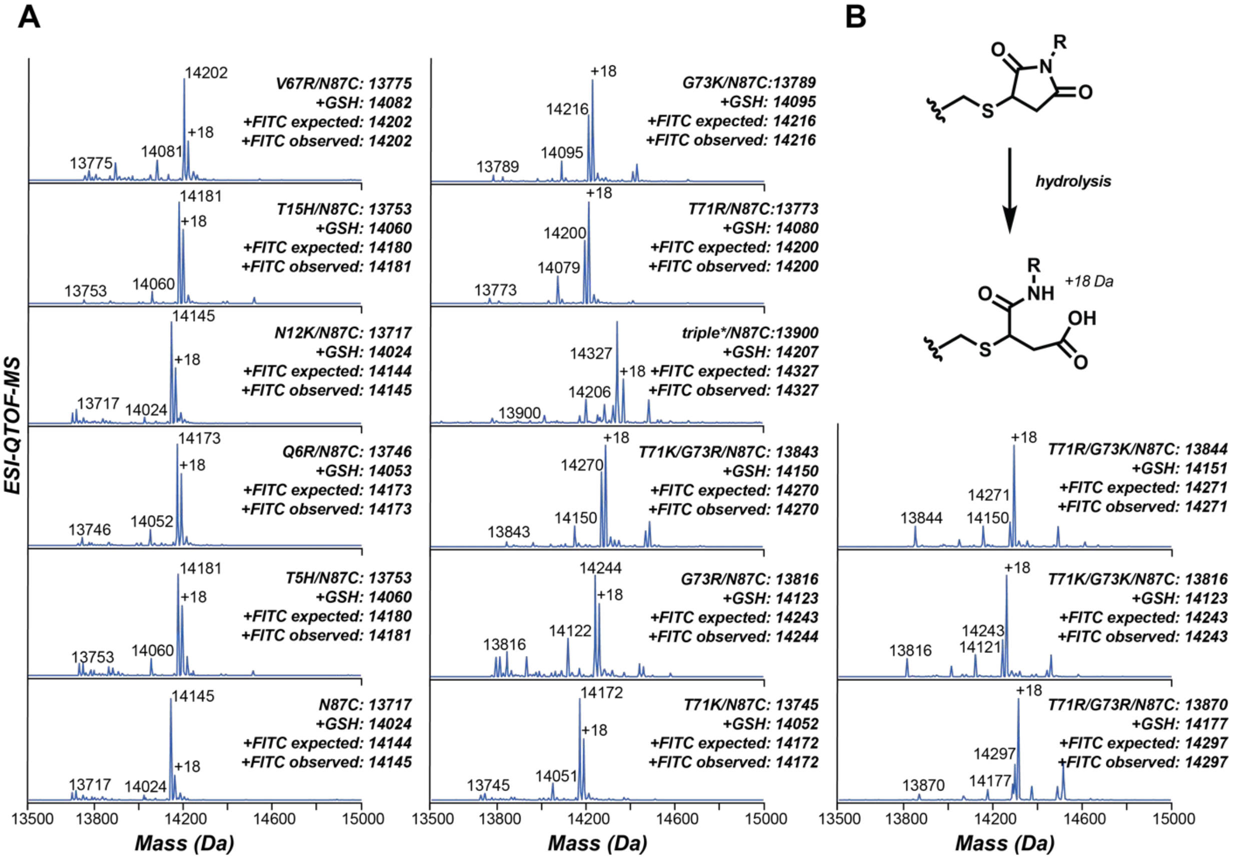

Figure S4. (A) LC-MS analysis of MS2-fluorescein conjugates. Each variant was modified 72-91% by fluorescein maleimide (FITC, +427 Da), and small amounts of a hydrolysis product (H_2O, +18 Da) and a glutathione (GSH, + 307 Da) addition product were observed. **(B)** Base-catalyzed hydrolysis product (+18 Da) of MS2-fluorescein maleimide conjugates observed in LC-MS traces.[1]

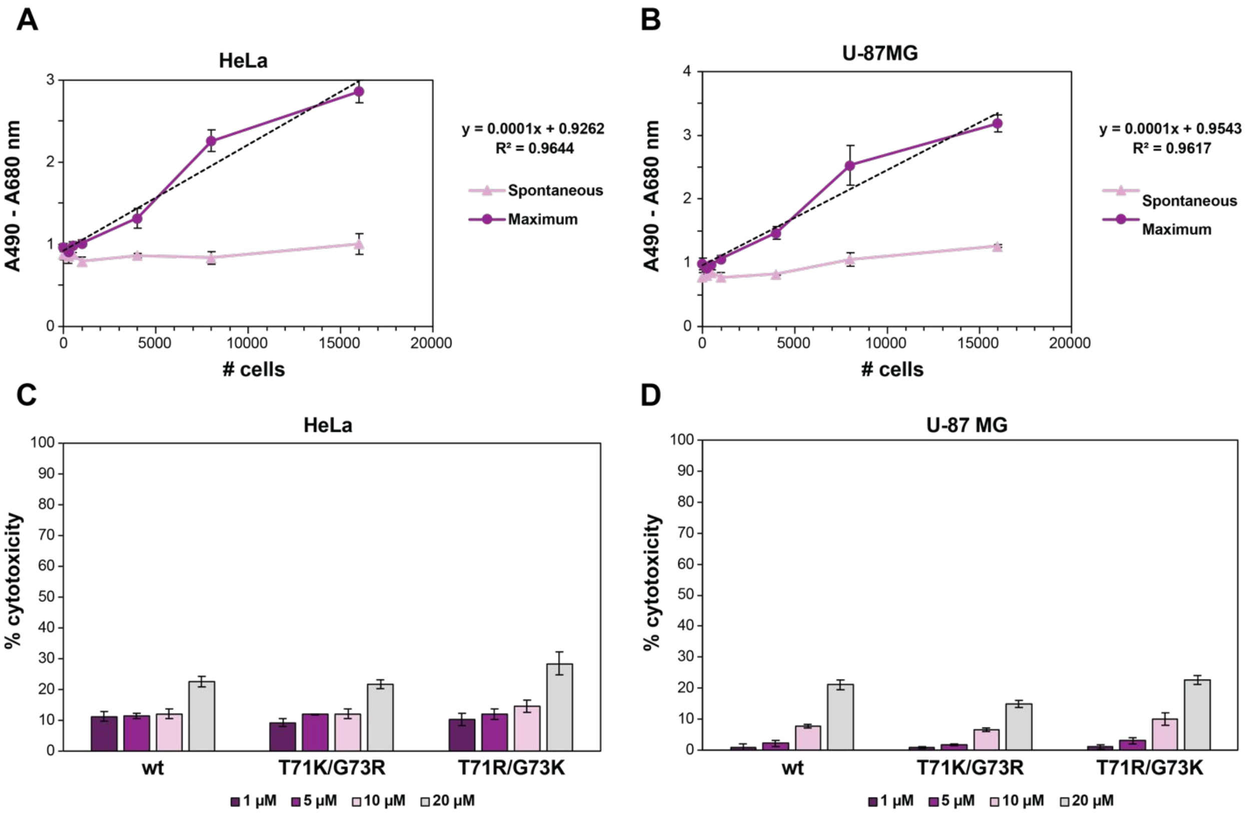

Figure S5. (A) LDH cytotoxicity assay standard curves for HeLa cells. **(B)** LDH cytotoxicity assay standard curves for U-87 MG cells. **(C)** LDH cytotoxicity assay for HeLa cells treated with 1, 5, 10, or 20 μM wt MS2, MS2 T71K/G73R, or MS2 T71R/G73K. Percent cytotoxicity shown as the mean of three biological replicates. Error bars show one standard deviation. **(D)** LDH cytotoxicity assay for U-87 MG cells treated with 1, 5, 10, or 20 μM wt MS2, MS2 T71K/G73R, or MS2 T71R/G73K. Percent cytotoxicity shown as the mean of three biological replicates. Error bars show one standard deviation.

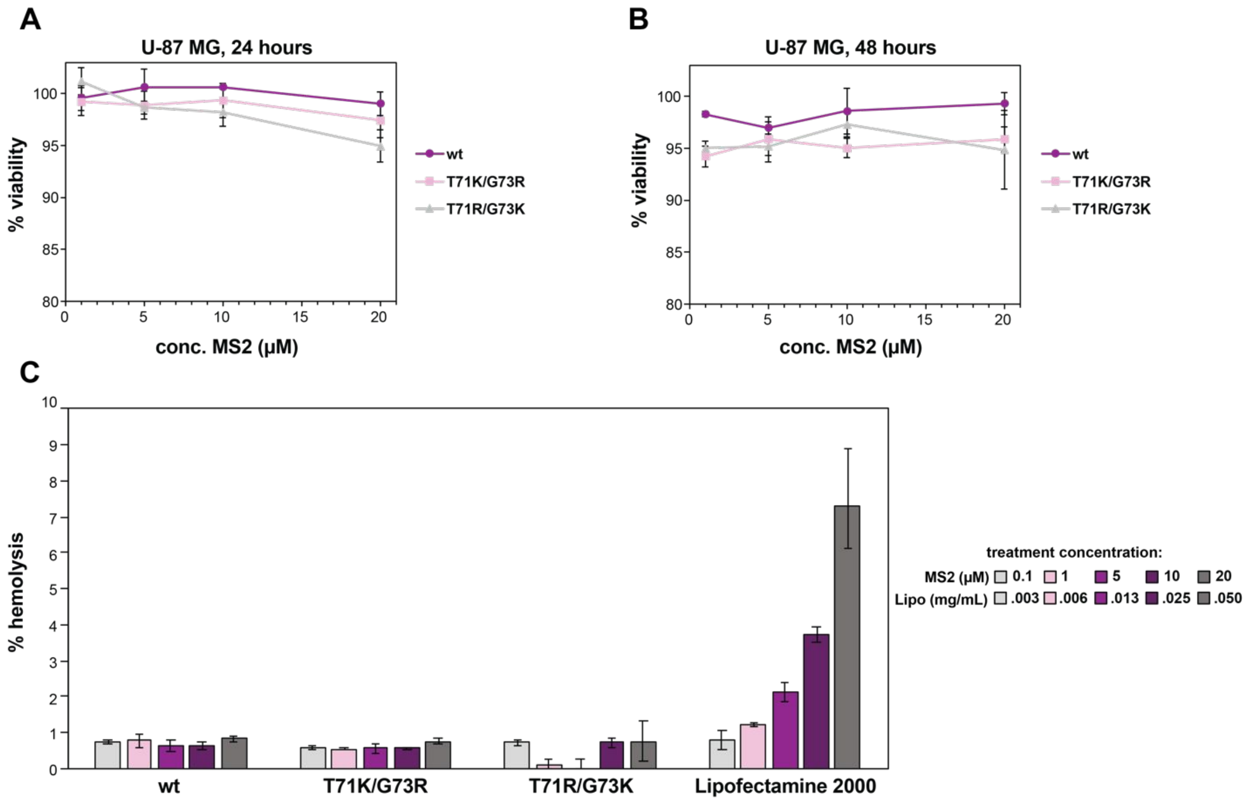

Figure S6. (A) MTS assay of 1, 5, 10, and 20 μM wt MS2 (purple circles), MS2 T71K/G73R (pink squares), or MS2 T71R/G73K (grey triangles) incubated with U-87 MG cells for 24 hours. Plot shows average % viability of three biological replicates. Error bars represent one standard deviation. **(B)** MTS assay of 1, 5, 10, and 20 μM wt MS2 (purple circles), MS2 T71K/G73R (pink squares), or MS2 T71R/G73K (grey triangles) incubated with U-87 MG cells for 48 hours. Plot shows average % viability of three biological replicates. Error bars represent one standard deviation. **(C)** Hemolysis assay measured after incubating 0.1-20 μM wt MS2, MS2 T71K/G73R, MS2 T71R/G73K, or .003-.050 mg/mL Lipofectamine 2000 with red blood cells for 3 hours at 37 °C. Percent hemolysis shown as the average of two biological replicates. Error bars show one standard deviation.

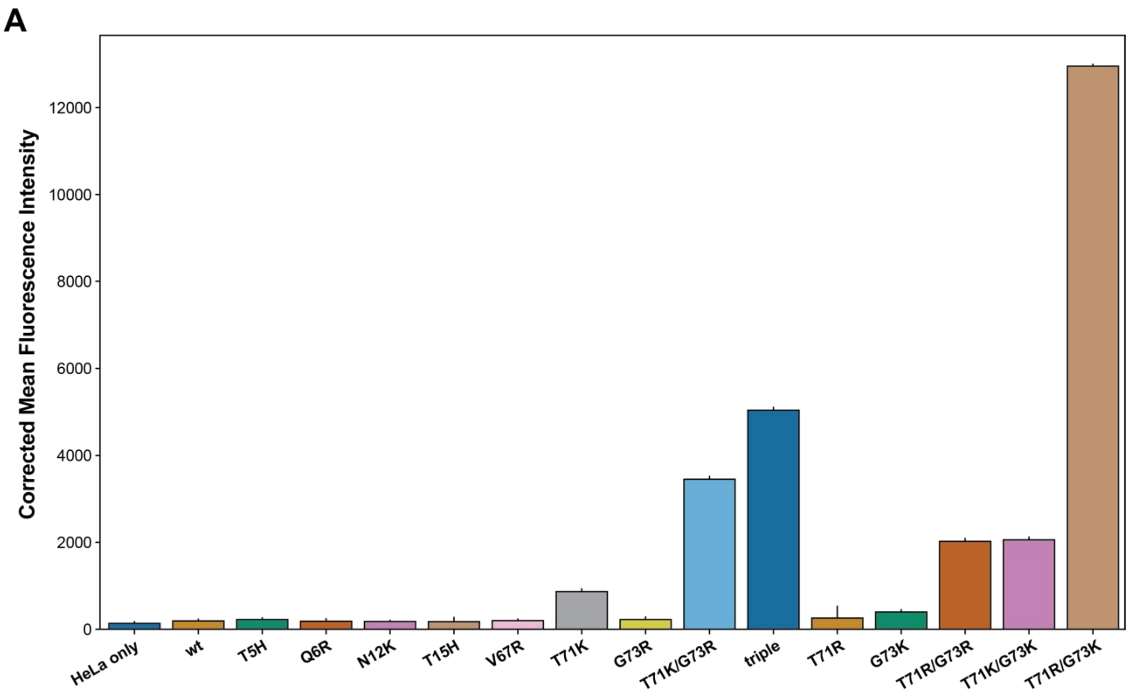

Figure S7. (A) Bar graphs showing corrected average fluorescence intensity values for MS2-fluorescein internalization into HeLa cells. Error bars show the coefficient of variation (CV).

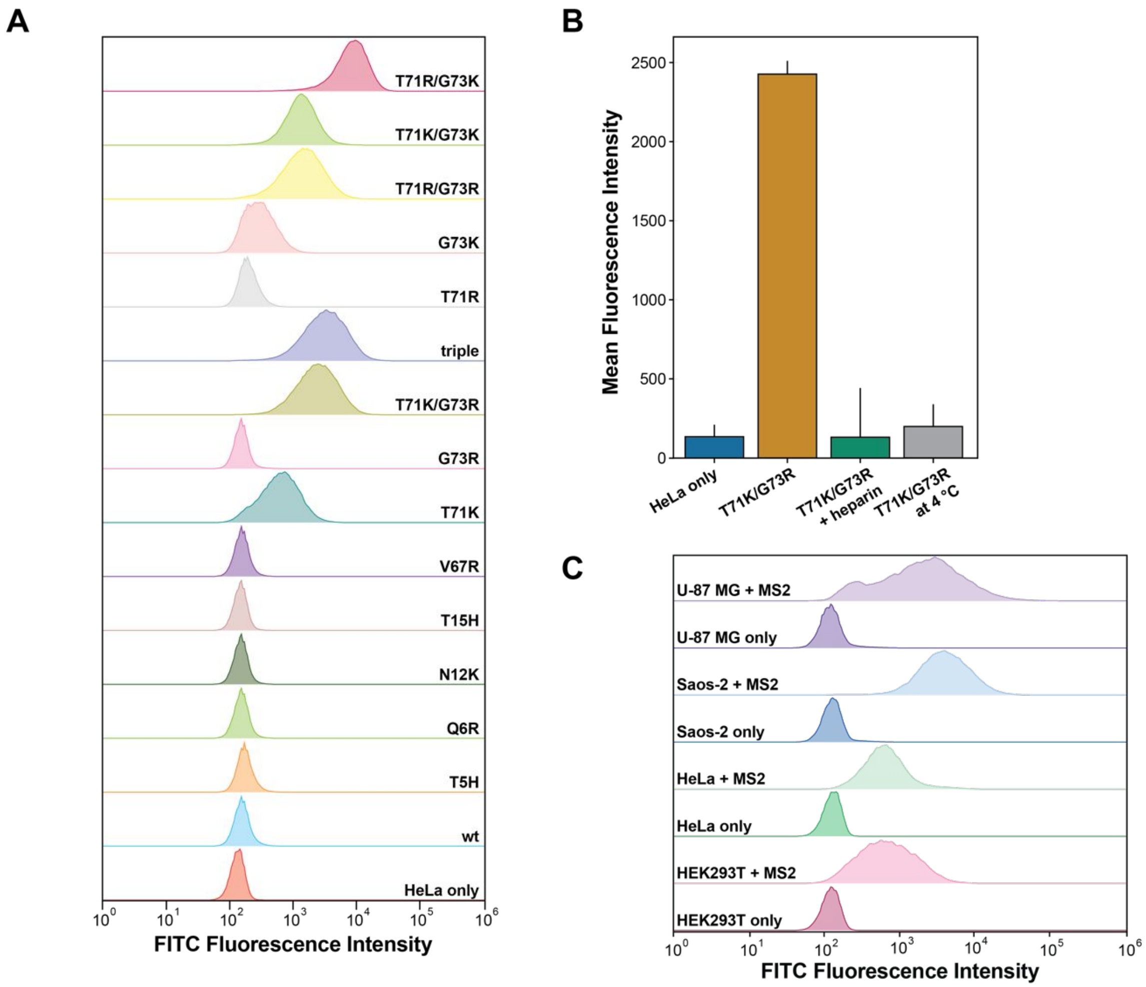

Figure S8. (A) Histograms showing uptake of MS2-fluorescein into HeLa cells. **(B)** Bar graphs showing median fluorescence intensity values for MS2 T71K/G73R-fluorescein internalization into HeLa cells in the presence of endocytosis inhibitors. Error bars show the coefficient of variation (CV). **(C)** Histograms showing uptake of MS2 T71K/G73R-fluorescein into HEK293T (pink), HeLa (green), Saos-2 (blue), and U-87 MG (purple) cells.

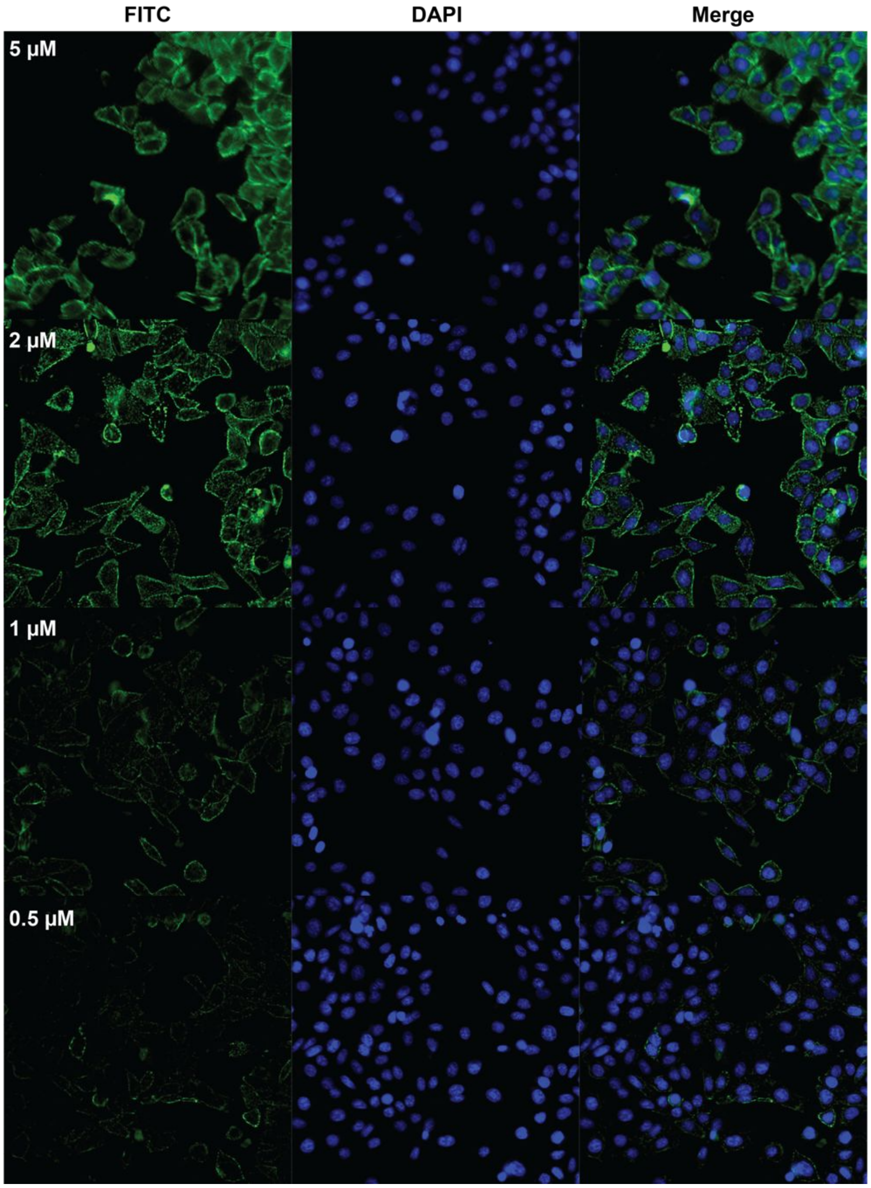

Figure S9. Fluorescence microscopy images of MS2-fluorescein internalization at different concentrations. From top to bottom: 5 µM monomers (28 nM capsids), 2 µM (11 nM capsids), 1 µM (5.5 nM capsids), 0.5 µM (2.8 nM).

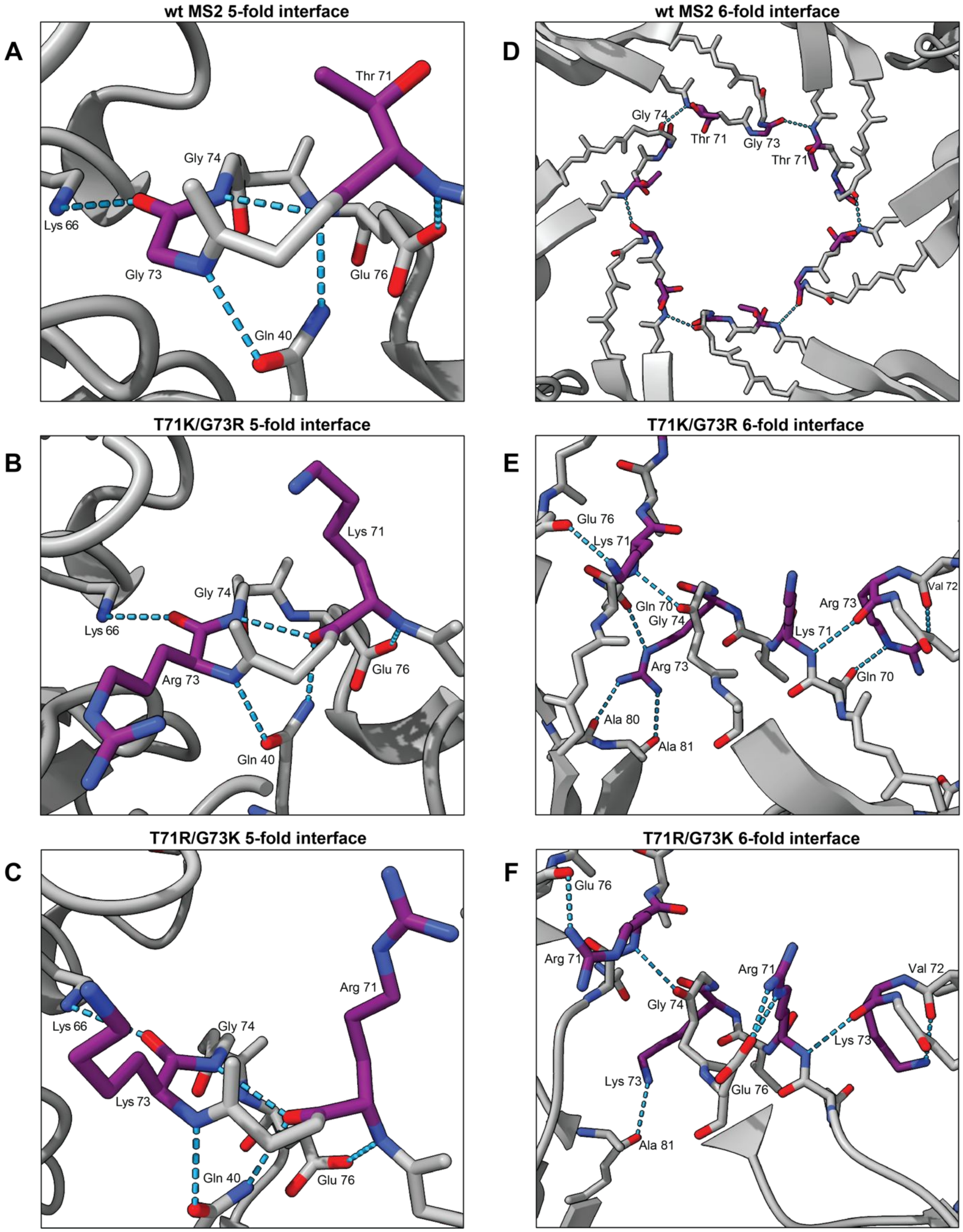

Figure S10. Comparison of hydrogen-bonding interactions at the 5- and 6-fold interfaces of MS2 CP variants. Residues 71 and 73 are colored in purple and other residues are colored in grey. Heteroatoms are colored red (oxygen) and blue (nitrogen) in residues of interest and hydrogen bonds are represented as light blue dashed lines.

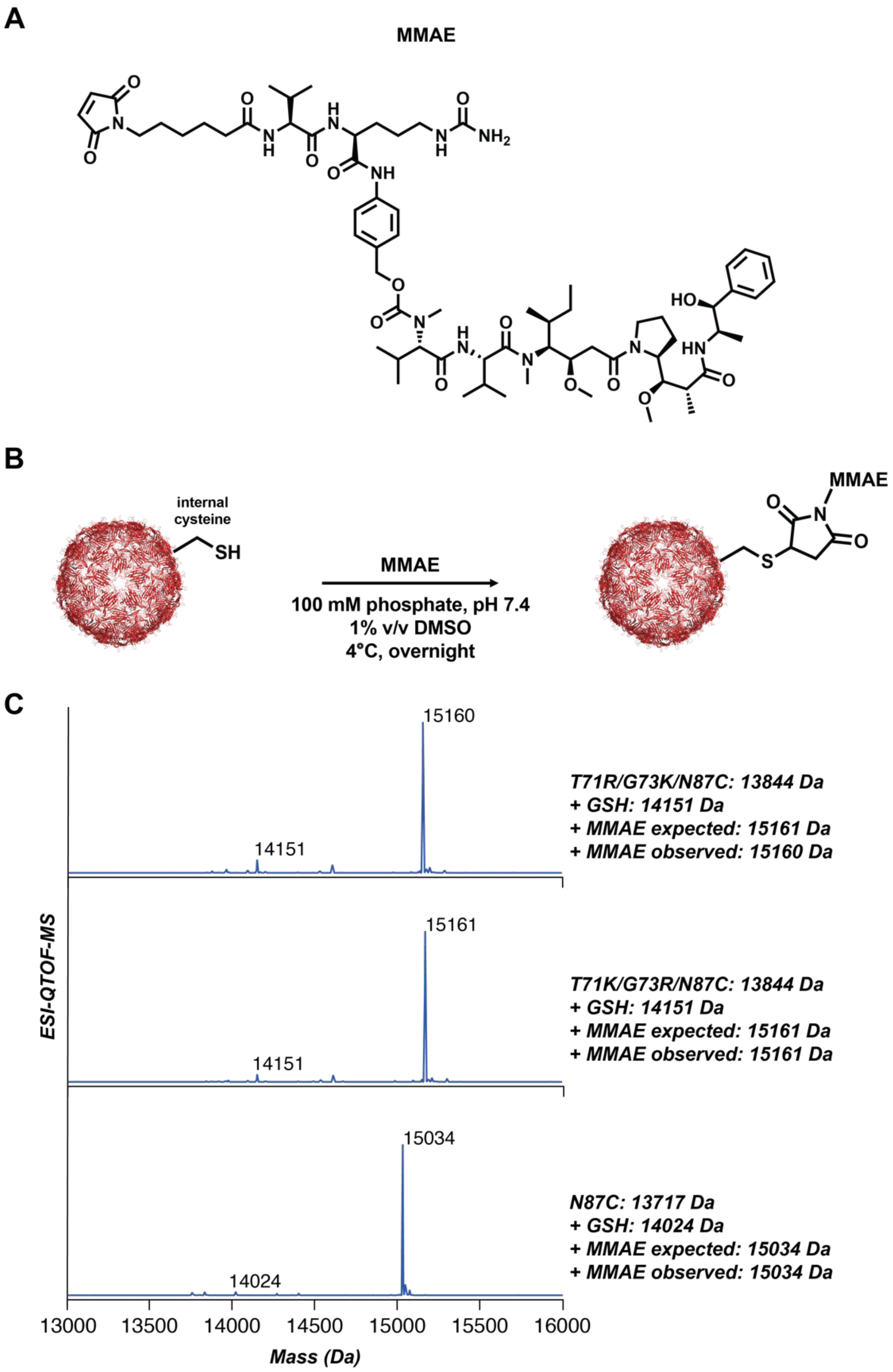

Figure S11. (A) Structure of maleimide-Val-Cit-PAB-MMAE (MMAE). **(B)** Reaction scheme describing maleimide-Val-Cit-PAB-MMAE coupling to the internal cysteine N87C of MS2 CP. **(C)** LC-MS analysis of MS2-MMAE conjugates. Each variant was modified 92-97% by maleimide-Val-Cit-PAB-MMAE (MMAE, +1317 Da), and small amounts of a glutathione (GSH, + 307 Da) addition product were observed.

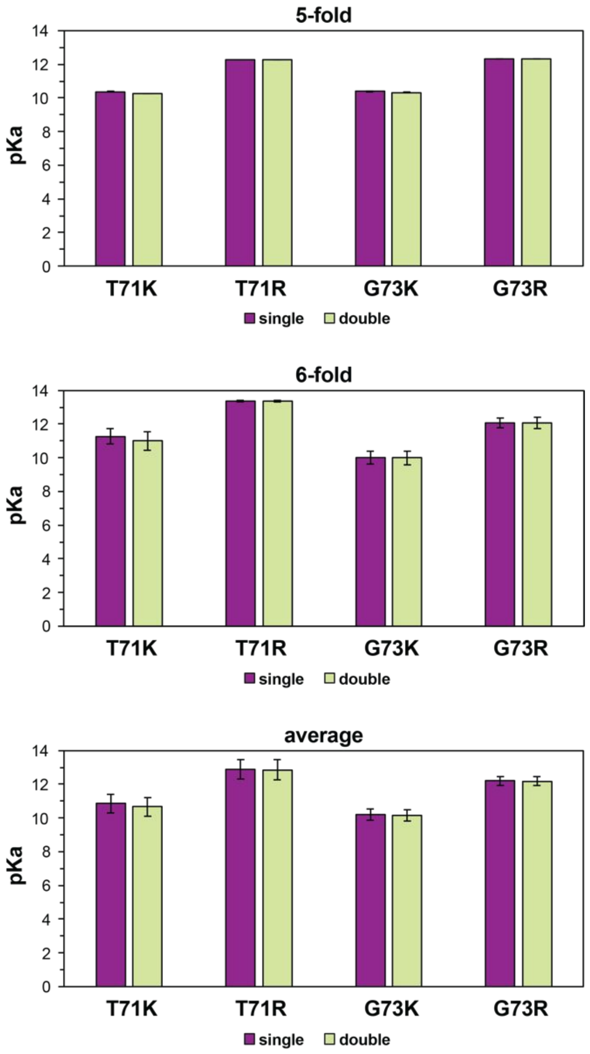

Figure S12. Comparison of calculated pK_as of residues at MS2 CP positions 71 and 73 between single and double mutants. Bar graph shows average single mutant pK_as (purple) and average double mutant pK_as (green). Error bars represent one standard deviation.

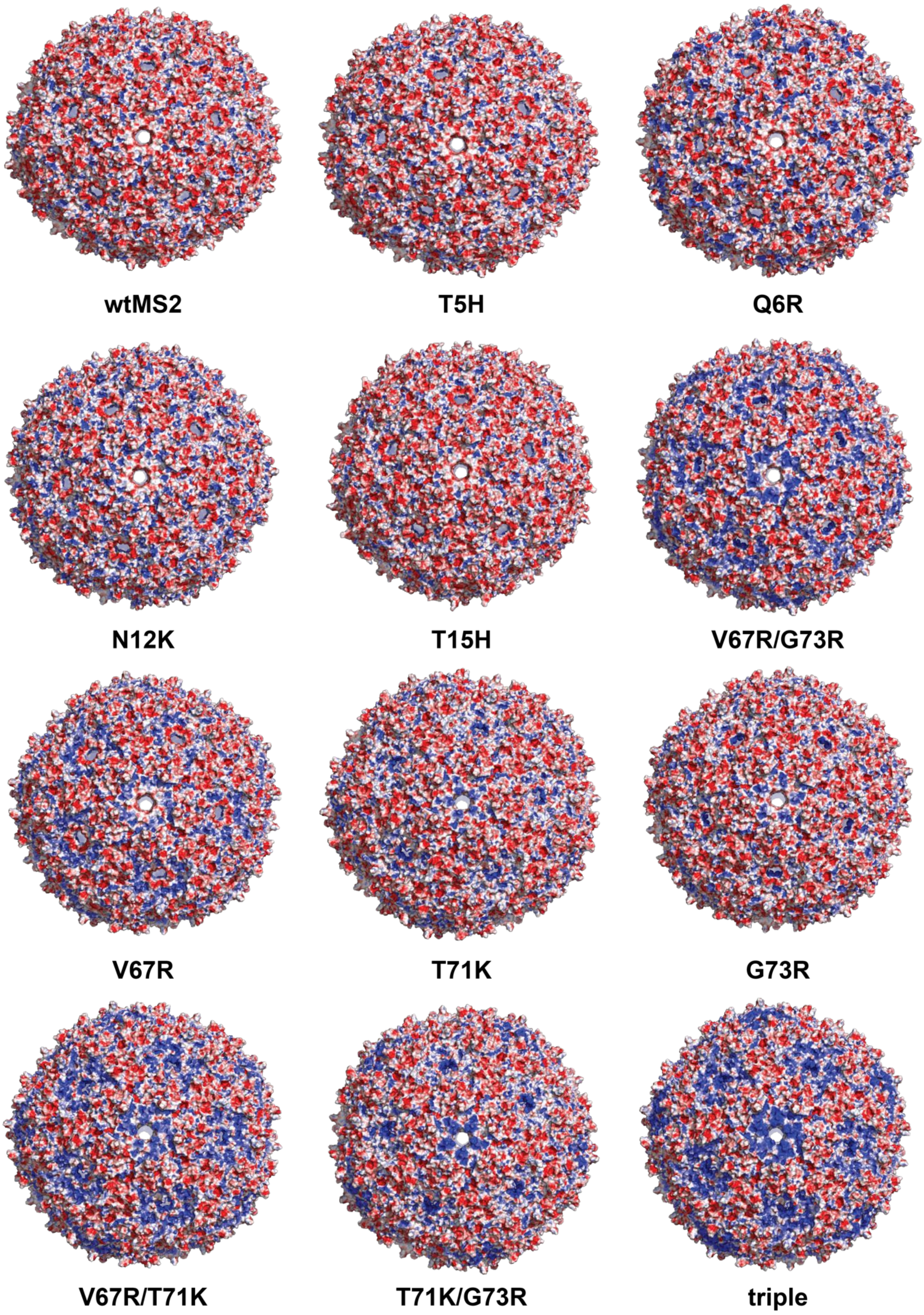
wtMS2
T5H
Q6R
N12K
T15H
V67R/G73R
V67R
T71K
G73R
V67R/T71K
T71K/G73R
triple

Figure S13. ABPS electrostatic potential maps of each MS2 CP variant. Positively charged (blue), negatively charged (red), and neutral (white) areas are mapped onto the surface of each capsid structure (PDB ID = 2MS2).

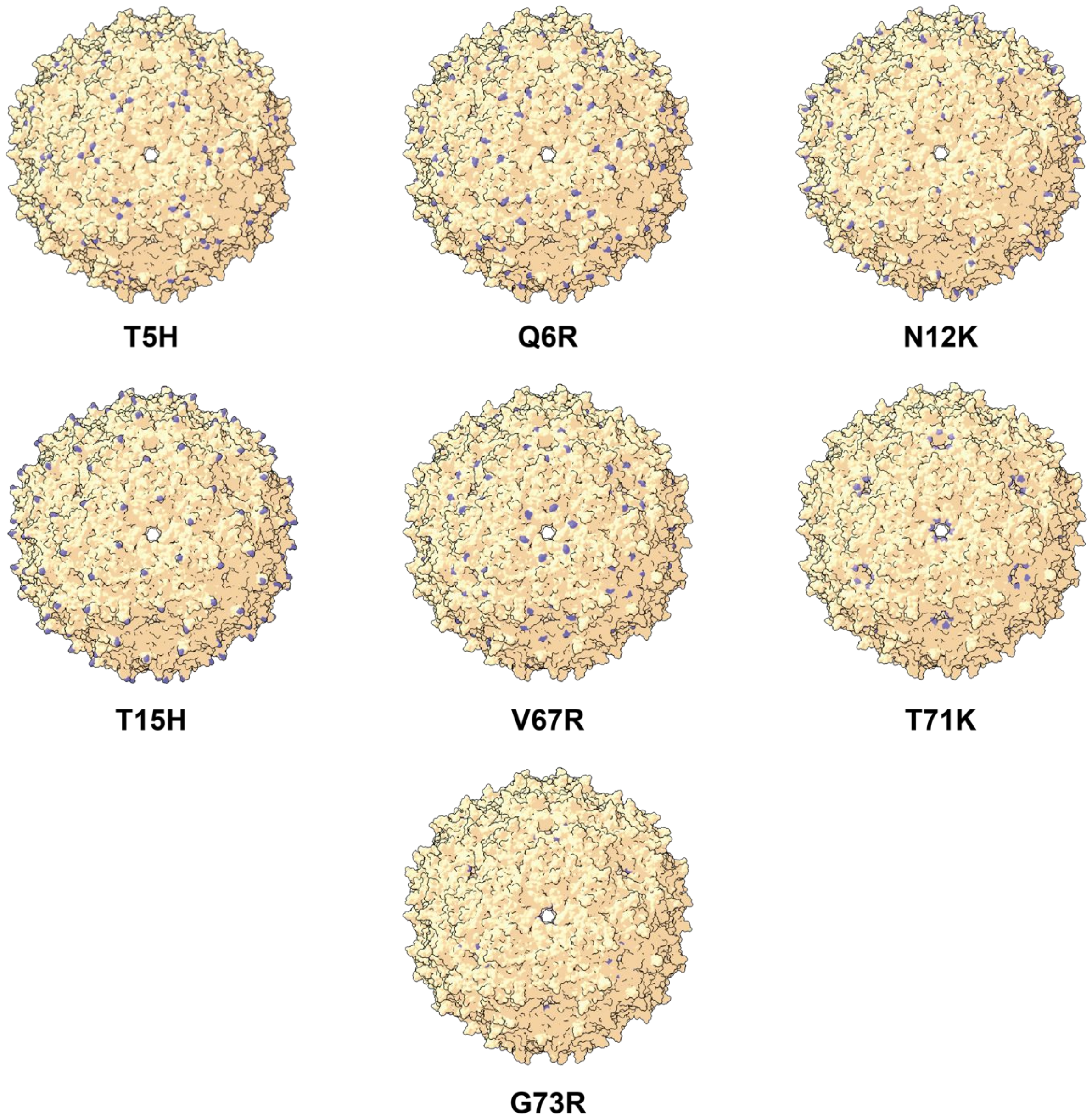

Figure S14. The locations of chosen positive charge mutations (blue) mapped onto assembled MS2 CP (PDB ID = 2MS2).

Supplementary Tables

Table S1. Masses (Da) of products obtained from MS2 CP variant fluorescein-maleimide labeling reactions determined by QTOF-ESI-MS. *Triple/N87C corresponds to MS2 CP variant V67R/T71K/G73R/N87C.

MS2 CP Variant	Unmodified Mass	+FITC Mass (Expected)	+FITC Mass (Observed)	+GSH Mass	% FITC Modification
N87C	13717	14144	14145	14024	87
T5H/N87C	13753	14180	14181	14060	83
Q6R/N87C	13746	14173	14173	14053	88
N12K/N87C	13717	14144	14145	14024	88
T15H/N87C	13753	14180	14181	14060	91
V67R/N87C	13775	14202	14202	14082	82
T71K/N87C	13745	14172	14172	14052	89
G73R/N87C	13816	14243	14244	14123	74
T71K/G73R/N87C	13843	14270	14270	14150	87
Triple*/N87C	13900	14327	14327	14207	81
T71R/N87C	13773	14200	14200	14080	84
G73K/N87C	13789	14216	14216	14096	87
T71R/G73R/N87C	13870	14297	14297	14177	90
T71K/G73K/N87C	13816	14243	14243	14123	77
T71R/G73K/N87C	13844	14271	14271	14151	72

Table S2. Mean flow cytometry fluorescence intensity values with coefficient of variation for MS2-fluorescein internalization into HeLa cells.

Treatment	Cell Count	Mean Fluorescence Intensity	Coefficient of Variation	Corrected Mean Fluorescence Intensity
HeLa Only	80755	137	50.2	137
N87C	72234	167	51.9	192
T5H/N87C	86323	185	53.3	223
Q6R/N87C	75705	162	71.1	184
N12K/N87C	85552	157	40.2	178
T15H/N87C	75718	158	112	174
V67R/N87C	86426	163	50.2	199
T71K/N87C	78429	771	77.4	866
G73R/N87C	86475	164	75.3	222
T71K/G73R	86845	3002	79.6	3451
Triple*/N87C	68515	4081	81.2	5038
T71R/N87C	82468	217	285	258
G73K/N87C	80755	345	64.3	397
T71R/G73R/N87C	78588	1820	81.1	2022
T71K/G73K/N87C	79780	1584	76.4	2057
T71R/G73K/N87C	82054	9326	56.1	12953

Table S3. Mean flow cytometry fluorescence intensity values with coefficient of variation for MS2 T71K/G73R-fluorescein internalization into HeLa, Saos-2, HEK293T, and U-87 MG cells.

Treatment	Cell Count	Median Fluorescence Intensity	Mean Fluorescence Intensity	Coefficient of Variation
HEK293T only	35110	124	131	152
HEK293T + MS2	39081	678	997	103
HeLa only	62664	130	134	72.3
HeLa + MS2	70525	638	872	113
Saos-2 only	34362	130	148	227
Saos-2 + MS2	54264	4033	5299	90.7
U-87 MG only	79218	122	141	135
U-87 MG + MS2	20497	2084	4078	191

Table S4. Mean flow cytometry fluorescence intensity values with coefficient of variation for MS2 T71K/G73R-fluorescein and MS2 T71R/G73K-fluorescein internalization into HeLa cells in the presence of endocytosis inhibitors.

Treatment	Cell Count	Median Fluorescence Intensity	Mean Fluorescence Intensity	Corrected MFI	Coefficient of Variation
HeLa only	18667	138	150	150	73.9
MS2 T71K/G73R only	6069	1481	1594	1881	75.4
T71K/G73R + heparin	5593	173	251	296	260
T71K/G73R + cyto D	9576	873	1058	1248	84.5
T71K/G73R + dynasore	8186	495	653	771	88.1
T71K/G73R + taxol	2163	1059	1265	1493	104
T71K/G73R + mβcd	27829	678	876	1034	101
T71K/G73R + 4 °C	53080	219	269	317	542
MS2 T71R/G73K only	49030	7568	7189	7189	71
T71R/G73K + heparin	51983	183	467	467	927
T71R/G73K + cyto D	27590	4974	5198	5198	87.8
T71R/G73K + dynasore	40303	4712	4928	4928	71.6
T71R/G73K + taxol	27968	7620	7150	7150	66.4
T71R/G73K + mβcd	29590	4301	4834	4834	85.9
T71R/G73K + 4 °C	57096	1845	2215	2215	81.6

Table S5. Isoelectric point (pI) estimates for MS2 CP variants calculated using Expasy, IPC2, and PROPKA.

MS2 CP Variant	Expasy pI (aa sequence)	IPC2 (aa sequence)	PROPKA pI (20mer)	PROPKA pI (12mer)
wt	7.93	6.78	8.57	8.51
T71K/G73R	8.87	7.91	8.75	8.70
T71R/G73K	8.87	7.92	8.75	8.69

Table S6. pK_a estimates for MS2 CP variants calculated using PROPKA.

MS2 CP Variant	Residue	20mer pK_a	12mer pK_a	Average pK_a
T71K/G73R	T71K	10.27	11.01	10.67
T71K/G73R	G73R	12.33	12.06	12.18
T71R/G73K	T71R	12.26	13.36	12.86
T71R/G73K	G73K	10.33	9.99	10.14
T71R	T71R	12.28	13.37	12.87
T71K	T71K	10.37	11.27	10.86
G73R	G73R	12.34	12.09	12.20
G73K	G73K	10.42	10.02	10.20

Table S7. Conformations of cationic amino acids at positions 71 and 73 of MS2 CP.

Mutation	5-fold conformation	6-fold conformation
T71K	capsid exterior	capsid exterior
G73R	capsid exterior	capsid interior
T71R	capsid exterior	capsid exterior
G73K	capsid exterior	capsid interior
T71K/G73R	T71K: capsid exterior<br>G73R: capsid exterior	T71K: capsid exterior<br>G73R: capsid interior
T71R/G73K	T71R: capsid exterior<br>G73K: capsid exterior	T71R: capsid exterior<br>G73K: capsid interior
T71R/G73R	T71R: capsid exterior<br>G73R: capsid exterior	T71R: capsid exterior<br>G73R: capsid interior
T71K/G73K	T71K: capsid exterior<br>G73K: capsid exterior	T71K: capsid exterior<br>G73K: capsid interior

Table S8. Apparent fitness scores (AFS), assembly scores, and thermostability scores for wild-type MS2, and all Arg and Lys single and double mutants at positions 71 and 73.[2]

MS2 Variant	AFS	Assembly	Thermostability
wild type	0.72	0.30	0.42
T71K	0.69	0.27	0.52
T71R	0.11	0.30	0.45
G73K	0.30	0.15	0.35
G73R	0.40	0.27	0.35
T71K/G73K	0.27	-0.13	-0.23
T71R/G73R	-0.21	-0.05	-0.40
T71K/G73R	0.37	-0.12	-0.29
T71R/G73K	-0.31	-0.1	-0.54

Table S9. Masses (Da) of products obtained from MS2 CP variant maleimide-Val-Cit-PAB-MMAE (MMAE) labeling reactions determined by QTOF-ESI-MS.

MS2 CP Variant	Unmodified Mass	+MMAE Mass (Expected)	+MMAE Mass (Observed)	+GSH Mass	% MMAE Modification
N87C	13717	15034	15034	14024	97
T71K/G73R/N87C	13844	15161	15161	14151	94
T71R/G73K/N87C	13844	15161	15160	14151	92

References

(1) Boyatzis, A. E.; Bringans, S. D.; Piggott, M. J.; Duong, M. N.; Lipscombe, R. J.; Arthur, P. G. Limiting the Hydrolysis and Oxidation of Maleimide–Peptide Adducts Improves Detection of Protein Thiol Oxidation. *J. Proteome Res.* **2017**, *16* (5), 2004–2015. https://doi.org/10.1021/acs.jproteome.6b01060.

(2) Hartman, E. C.; Lobba, M. J.; Favor, A. H.; Robinson, S. A.; Francis, M. B.; Tullman-Ercek, D. Experimental Evaluation of Coevolution in a Self-Assembling Particle. *Biochemistry* **2019**, *58* (11), 1527–1538. https://doi.org/10.1021/acs.biochem.8b00948.
